# Network reorganisation following anterior temporal lobe resection and relation with post-surgery seizure relapse: A longitudinal study

**DOI:** 10.1016/j.nicl.2020.102320

**Published:** 2020-06-26

**Authors:** Nádia Moreira da Silva, Rob Forsyth, Andrew McEvoy, Anna Miserocchi, Jane de Tisi, Sjoerd B. Vos, Gavin P. Winston, John Duncan, Yujiang Wang, Peter N. Taylor

**Affiliations:** a CNNP lab^1^, Interdisciplinary Complex Systems Group, School of Computing, Newcastle University, Newcastle upon Tyne, United Kingdom; b Translational and Clinical Research Institute, Newcastle University, Newcastle upon Tyne, United Kingdom; c NIHR University College London Hospitals Biomedical Research Centre, UCL Queen Square Institute of Neurology, Queen Square, London, United Kingdom; d Centre for Medical Image Computing, University College London, London, United Kingdom; e Epilepsy Society MRI Unit, Chalfont St Peter, United Kingdom; f Department of Medicine, Division of Neurology, Queen’s University, Kingston, Canada

**Keywords:** Temporal Lobe Epilepsy, Longitudinal study, Seizure-freedom

## Abstract

**Objective:**

To characterise temporal lobe epilepsy (TLE) surgery-induced changes in brain network properties, as measured using diffusion weighted MRI, and investigate their association with postoperative seizure-freedom.

**Methods:**

For 48 patients who underwent anterior temporal lobe resection, diffusion weighted MRI was acquired pre-operatively, 3–4 months post-operatively (N = 48), and again 12 months post-operatively (N = 13). Data for 17 controls were also acquired over the same period. After registering all subjects to a common space, we performed two complementary analyses of the subjects’ quantitative anisotropy (QA) maps. 1) A connectometry analysis which is sensitive to changes in subsections of fasciculi. 2) A graph theory approach which integrates connectivity information across the wider brain network.

**Results:**

We found significant postoperative alterations in QA in patients relative to controls measured over the same period. Reductions were primarily located in the uncinate fasciculus and inferior fronto-occipital fasciculus ipsilaterally for all patients. Larger reductions were associated with postoperative seizure-freedom in left TLE. Increased QA was mainly located in corona radiata and corticopontine tracts. Graph theoretic analysis revealed widespread increases in nodal betweenness centrality, which were not associated with patient outcomes.

**Conclusion:**

Substantial alterations in QA occur in the months after epilepsy surgery, suggesting Wallerian degeneration and strengthening of specific white matter tracts. Greater reductions in QA were related to postoperative seizure freedom in left TLE.

## Highlights

• Diffusion changes assessed at two time points following epilepsy surgery.
• Graph theory and connectometry revealed substantial longitudinal diffusion changes.
• Changes were found beyond the site of resection.
• Postoperative seizure freedom associated with longitudinal structural changes.

## 1 Introduction

Temporal lobe epilepsy (TLE) is the most common form of drug-refractory focal epilepsy. For patients with intractable TLE, surgery can be a viable treatment option. In individuals with TLE, an anterior temporal lobe resection (ATLR) leads to seizure freedom in 50–80% after at least 1 year of follow-up (Spencer and Huh, 2008, de Tisi et al., 2011). An ATLR involves the removal of most of the amygdala, hippocampus as far posterior as the widest part of the brainstem, entorhinal, and other temporal neocortical areas. White matter pathways including the uncinate fasciculus, inferior longitudinal fasciculus and optic radiation may be transected.

Microstructural properties of subcortical white matter pathways can be measured *in vivo* using diffusion MRI. Many previous studies have investigated white matter properties in TLE patients and found differences relative to controls (Winston et al., 2014, Li et al., 2019, Keller et al., 2017, Schoene-Bake et al., 2009, Elliott et al., 2018, Gross et al., 2006, Riley et al., 2010, Taylor et al., 2015). Some studies have additionally related pre-surgical white matter properties to post-surgical seizure-freedom. Alizadeh et al., (2019) showed an association between seizure-recurrence and tract density. Furthermore, Keller et al., 2017, Taylor et al., 2018 demonstrated that preoperative white matter alterations that were later resected were associated with post-operative outcomes. Both studies investigated associations with postoperative seizure freedom, but did not use postoperative diffusion MRI.

Some studies have investigated postoperative white matter properties using diffusion MRI. Concha et al. (2007) showed reduced postoperative fractional anisotropy (FA) in a cohort of eight seizure-free patients in the fornix, cingulum, and other areas. McDonald et al. (2010) demonstrated, in seven seizure-free patients, reduced postoperative FA in the uncinate fasciculus, inferior longitudinal fasciculus, and fornix. Other studies also found ipsilateral reductions in FA, likely due to Wallerian degeneration (Yogarajah et al., 2010, Nguyen et al., 2011, Faber et al., 2013, Liu et al., 2013, Pustina et al., 2014, Winston et al., 2014). Although several of those studies have related surgery-induced changes to neuropsychological consequences, such as visual field deficits (McDonald et al., 2010) and verbal fluency (Pustina et al., 2014, Yogarajah et al., 2010), none have investigated associations with postoperative seizure-freedom.

In this study we characterised surgery induced white matter changes in a longitudinal cohort of 48 patients who underwent ATLR and addressed the following questions: What is the impact of surgery within white matter fasciculi? Do white matter changes drive alterations in network topology? Do these changes to white matter fasciculi and/or network topology relate to seizure-freedom?

## 2 Materials and Methods

### 2.1 Participants

We studied 48 patients who underwent ATLR at the National Hospital of Neurology and Neurosurgery (NHNN), London, United Kingdom and 17 healthy controls, with no significant medical history of neurological or psychiatric problems. Twenty-two of the 48 patients had right ATLR and 26 on the left. All patients had resection of the majority of the amygdala and hippocampus as far posteriorly as the widest part of the brainstem, using a modified Spencer ATLR (Spencer et al., 1984), without complication. All surgeries went according to plan, as verified by post-operative MRI at 3–4 months following surgery. Patients were followed up for 2–5 years and at every 12 months surgical outcome was recorded according to the ILAE scale (Wieser et al., 2001). Two-year ILAE outcomes were available for all patients, with three-, four- and five-year outcomes available for 46, 40 and 34 patients respectively. Eleven patients with left TLE and 5 with right TLE had remained entirely seizure free (ILAE 1) throughout a 5-year follow-up period. Eleven patients with left TLE and 12 with right TLE had postoperative seizures at least once during their follow-up period of 2 to 5 years.

A $\chi^{2}$ test and two-tailed non-parametric Wilcoxon ranksum test confirmed differences in sex but not in age between controls and patients. Differences were regressed out in further analysis. Tables 1 and S1-S2 summarise patient details.

**Table 1:** Demographic and clinical data of patients and controls with imaging available 3 months after surgery.

	Left TLE	Right TLE	Controls	Significance
*Subjects (n)*	26	22	17	–
*Sex (Male/Female)*	13/13	2/20	9/8	Chi2 _(Left TLE; Controls)_ = 0.035 p _(Left TLE; Controls)_ = 0.850 Chi2 _(Right TLE; Controls)_ = 9.106 **p _(Right TLE; Controls)_ = 0.003**
*Age at baseline scan*	35.86 ± 9.57	39.83 ± 12.26	40.06 ± 11.06	t _(Left TLE; Controls)_ = -1.318 p _(Left TLE; Controls)_ = 0.195 t _(Right TLE; Controls)_ = -0.060 p _(Right TLE; Controls)_ = 0.953
*Onset*	16.19 ± 10.93	14.93 ± 10.75	–	–
*Age at surgery*	36.52 ± 9.62	41.00 ± 12.42	–	–
*Epilepsy Duration*	20.33 ± 13.67	26.07 ± 13.35	–	–
*Status Epilepticus (n)*	3	1	–	–
*Secondary generalised seizures (n)*	20	14	–	–
*Hippocampal Sclerosis (n)*	18	11	–	–
*Seizure-Free for 5 years (n)*	11	5	–	–
*Seizure recurrence (n)*	11	12	–	–

The study was approved by the NHNN and the Institute of Neurology Joint Research Ethics Committee, and written informed consent was obtained from all subjects.

### 2.2 MRI acquisition and imaging processing

For each patient in this study, T1-weighted structural (sMRI) and diffusion weighted (dMRI) data were acquired pre-operatively and 3–4 months post-operatively. A subset of these patients (N = 13) were scanned again 12-months following surgery. Controls also underwent sMRI and dMRI at three time points: baseline (N = 17) followed by 3 months (N = 17) and 12 months (N = 16) later. Control data were used as a normative measure for the longitudinal changes obtained from patients over 3–4 months after surgery and between and 12 months after surgery. Detailed imaging protocols are described in Supplementary Methods. Postoperative sMRI, linearly registered to preoperative sMRI using FSL FLIRT, was used to accurately delineate the resected tissue in patients. More details on how the resection mask was drawn for these subjects are available in Taylor et al. (2018).

Diffusion data were reconstructed using generalized q-sampling imaging with a diffusion sampling length ratio of 1.2 and CSF calibration, as in our previous study using DSI Studio (http://dsi-studio.labsolver.org) (Taylor et al., 2018). The deterministic fibre tracking algorithm was used, allowing for crossing fibres within voxels, and to reduce false positive connections, which can critically alter graph metrics in comparison with false negative connections (Zalesky et al., 2016). The diffusion reconstruction was performed in the MNI ICBM2009c template space. FA images were also obtained using dtifit and all subjects’ preoperative FA data was aligned to the FMRIB58_FA standard space template using a nonlinear registration tool FNIRT. Each subjects’ postoperative FA image was co-registered to its preoperative FA image using the linear registration tool FLIRT. Since there are slight anatomical differences between MNI ICBM2009c and the 6th generation of 152MNI space available in FSL (Mai and Majtanik, 2017), we applied a non-linear registration between MNI templates. The warps derived from both analyses were combined and applied to the original resection masks and FA images as in our previous study (Yogarajah et al., 2010). QA is related to fibre volume fraction (Yeh et al., 2013b) and is highly correlated with other measures such as FA and mean diffusivity (MD). Although it is less commonly used, QA has important advantages. Phantom studies have shown it to be less susceptible to partial volume effects, and artefactual reductions in areas of crossing fibres (Yeh et al., 2013b). Visual assessment of registration quality was conducted for all images. The resection masks from all patients aligned into the MNI ICBM 2009c template were binarized into a single mask using FSL. We performed this step for left and right TLE separately. The overlaid resection masks were used to delineate resected tissue in further analyses. To quantify the similarity of resected tissue across TLE patients, we computed Dice overlap (Moreira da Silva et al., 2017, Traynor et al., 2010) separately for left and right TLE patients. Furthermore, we investigated if resection volume was related to outcomes of seizure freedom.

### 2.3 Analysis of post-operative diffusion changes

We performed two complementary analyses to study post-operative changes in diffusion MRI. Connectometry allows characterisation of specific focal and spatial structural changes within subsections of tracts which may drive alterations in brain connectivity or network topology (Yeh et al., 2016, Yeh et al., 2018). Graph theory allows quantification of those alterations in network topology locally; by measuring properties of grey matter regions, and globally; by measuring properties of the network as a whole (Yeh et al., 2016).

#### 2.3.1 Connectometry: diffusion changes in subsections of white matter bundles

Connectometry is sensitive to changes in *subsections* of tracts, which otherwise could be hidden by conventional atlas-based or connectome approaches (Yeh et al., 2013a, Yeh et al., 2013b, Yeh et al., 2016, Faraji et al., 2015, Sinha et al., 2019). These conventional approaches compute mean diffusion measures along the entire length of white matter tracts (Supplementary Methods: Atlas-based approach). Connectometry is robust to crossing fibres and, while identifying diffusion alterations, does not depend on tractography, which has limited reliability near the grey matter targets. These are two main advantages in comparison to other methods for characterisation of focal changes within tracts (Yeh et al., 2016). Thus, we studied the structural alterations in subsections of tracts using diffusion MRI connectometry to compare patient and control groups and patient association with postoperative outcome.

First, we created two connectometry databases comprising controls and left or right TLE patients (26 left TLE patients and 17 controls; 22 right TLE patients and 17 controls) with QA as the index of interest. The default Human Connectome Project HCP1021 template was used from which the local fibre directions were sampled to create the local connectome matrix.

Secondly, we ran the group connectometry analysis in DSI studio to correlate the local connectome matrix with subject category (patient or control) in a multiple linear regression model, while controlling for age and sex. We constrained the analysis to the non-resected area to estimate only regional differences of the local connectome within non-resected tissue. Thus, overlaid resection masks were used as region of avoidance to discard any voxel that was resected across any patient. We employed this analysis separately for left and right TLE patients. To compare patient and control groups, we applied the same approach to controls using the left and right overlaid resection masks separately.

We ran connectometry analysis with different t-score thresholds to select local connectomes (T-score: 1, 2 and 3) at different significance levels (FDR: 0.05, 0.075 and 0.1) (Wang et al., 2019). Higher values of t-score give more specific and confirmatory results, whereas lower values give more sensitive results, which are useful for exploratory studies. Although low FDR values might filter most of the false discoveries, they might not detect subtle differences (Yeh et al., 2013a). Thus, a wider range was employed for exploratory purposes.

At each point on this two dimensional grid, local connectomes for every seed voxel under analysis are inspected for positive and negative associations with subject category. The local connectomes were tracked using a deterministic fibre tracking algorithm (Yeh et al., 2013a, Yeh et al., 2013b), track trimming was iterated once, and the seed count was set to 10,000. These are the default settings for fibre tracking in connectometry analysis from the 9 September 2018 version of DSI Studio. Connectometry analysis controls for false discoveries by comparing the positive and negative associations of QA with a null distribution obtained by randomly permuting the group labels. Further details of this procedure are available in the section “Local connectomes and their statistical inference” in Yeh et al., (2016) and also in the method section of Yeh et al., 2013a, Yeh et al., 2013b. In our study, we used the default permutation count of 2000.

Thirdly, we quantified the output of the connectometry analysis at each point of the two-dimensional grid. The subsections of tracts found to be significantly different between patients and controls were grouped as in the population-average atlas of the connectome (Yeh et al., 2018). This atlas identifies 550,000 white-matter tracts verified manually by experienced neuroanatomists, which are grouped in 80 main bundles. Cranial nerves, cerebellum and brainstem bundles were not considered in this study. Thus, subsections located in the same bundle as in the atlas were grouped (Supplementary Methods: Group subsections from connectometry). Then, for each group, we computed the mean QA for each subject and timepoint. Effects of age and sex were regressed out using fitlm function in MATLAB.

Investigating which grey matter regions these subsections connect to may improve our understanding of patterns of degeneration and plasticity following ATLR. Thus, tracts in which subsections were found to be significantly different by connectometry were found by identifying the nearest tract in the connectome atlas to each subsection. Only tracts within 1 mm were included. After estimating the entire tracts, we quantified a connectivity matrix using AAL parcellation scheme. Voxels of the AAL regions overlapping with the overlaid resection mask were removed. This was conducted separately for left and right TLE patients.

In order to investigate how changes within tracts were related with postoperative outcomes, we split patients into seizure-free or seizure-recurrence groups and repeated the previous steps in this analysis. Therefore, we created a database with patient data and used their postoperative outcome as the variable of study in the multiple linear regression model.

To support the interpretation of QA results, the previous steps were repeated using FA as index of interest.

#### 2.3.2 Graph theory: changes in network topology

To study how alterations in the tracts drive changes in the network topology, we used graph theory to estimate longitudinal changes in network metrics between patients and controls. We characterised the changes in network topology locally, by studying different grey matter regions (nodes) and globally, by studying the overall differences in the whole brain.

Tracts from the population-average atlas of the connectome (Yeh et al., 2018) were projected to each individuals’ QA image using DSI Studio. The mean QA along the tracts connecting each pair of regions of the AAL atlas (Tzourio-Mazoyer et al., 2002) was estimated and saved in a connectivity matrix for each individual and timepoint. Voxels of the AAL regions overlapping with the resection mask were removed. This was conducted separately for left and right TLE patients.

Network metrics, including node strength, clustering coefficient, eigenvector and betweenness centrality, were estimated from the connectivity matrices to quantify network properties (Rubinov and Sporns, 2010). Effects of age and sex were regressed out using fitlm function in MATLAB. A detailed description of all the metrics used is provided in the Supplementary Table S3.

After calculating network metrics from the connectivity matrix of each participant at each timepoint, we computed the longitudinal change in graph metric (K) over 3–4 months:

(eg. $K\;\mathrm{left}\;{\mathrm{thalamus}}_{3-4months\;postop}-K\;\mathrm{left}\;t{\mathrm{halamus}}_{\mathrm{baseline}}$)

and over the following 8–9 months:

(eg. $K\;\mathrm{left}\;{\mathrm{thalamus}}_{12months\;postop}-K\;\mathrm{left}\;{\mathrm{thalamus}}_{3-4months\;postop}$).

For visualisation, we computed the z-score of the longitudinal changes in patient group as the number of standard deviations away from the mean, where the standard deviation and mean were obtained from the control distribution. High $\left|z-score\right|$ indicates high deviation of network properties from normality. To observe if there were statistical differences over the same time period between patients and controls, we used inference statistical tests. These differences might be associated with (1) the impact of surgical resection on the network and (2) plasticity of white matter tracts following surgery. We run two further analyses to distinguish both mechanisms. The first analysis is similar to that described in Taylor et al., 2018; we inferred the impact of surgical resection on network topology using preoperative data only. Thus, we computed the expected change on network metrics using the difference between the preoperative network without the resected tracts and the preoperative network with all tracts. For ease of interpretation we will refer to this analysis as the null model. For the second analysis, we computed for all patients the difference between the postoperative network and the preoperative network with all tracts. This difference represents both the impact of surgical resection on the network *and* plasticity/degeneration following it. For ease of interpretation, we will refer to this analysis as an observed model since the network is generated from the empirically observed postoperative data. By comparing both analyses we are able to distinguish the postoperative changes in network metrics due to the impact of surgical resection from changes due to reorganisation of the connectome.

To study the association of postoperative outcome with network metrics, patients were split into seizure-free and seizure-recurrence groups. The longitudinal changes in network metrics between both groups were compared using inference statistical tests.

### 2.4 Statistical analysis

Inferential statistical tests were used to compare longitudinal changes between patient and control groups and subclasses in patients based on their postoperative outcome. We applied nonparametric Wilcoxon rank-sum test to compare longitudinal changes in network metrics between groups and applied an FDR (Benjamini-Hochberg) correction at q = 0.05.

Connectometry uses permutation tests to obtain a null distribution of the connectometry findings and estimate a FDR value. Further details are available in Yeh et al., 2013a, Yeh et al., 2013b, Yeh et al., 2016.

## 3 Results

**Fig. 1:**
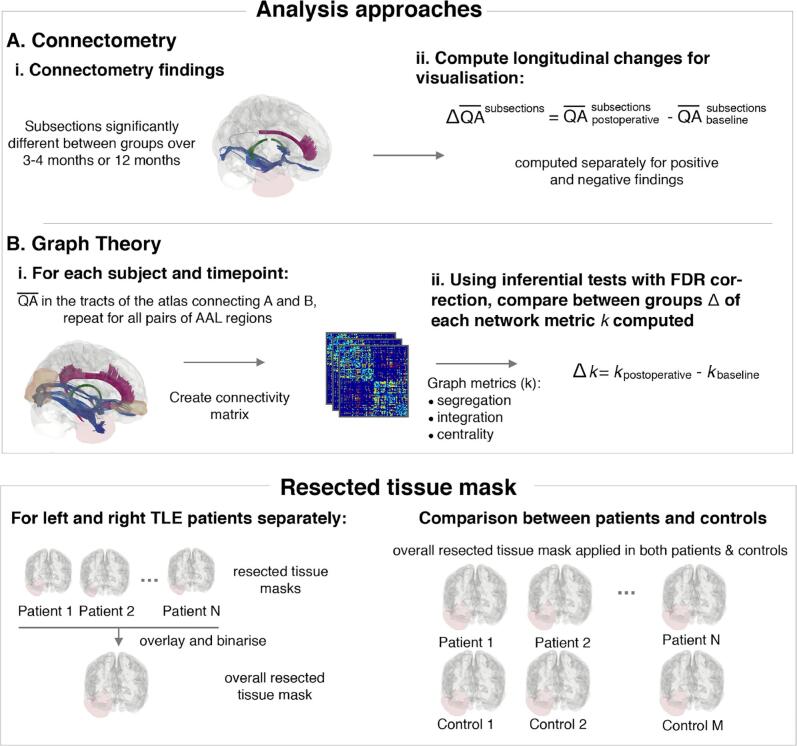
**Processing pipeline.** To compare patient and control groups or patients with different postoperative outcome, two complementary analyses were performed. **(A)** A connectometry analysis which is sensitive to changes in subsections of bundles was employed to investigate local differences within bundles: Connnectometry identified subsections within bundles in the whole brain where QA changes over time were significantly different between groups. **(B)** A graph theory approach was applied to integrate connectivity across the wider brain network: To produce connectivity matrices, mean QA along the bundles of the connectome atlas connecting each pair of AAL regions was computed for each subject. Graph theory was used to compare changes in network properties over time. Resected tissue mask: The resected tissue masks of all patients were overlaid in a single mask to be used as region of avoidance in (A) and (B). When comparing patient and control groups, that single mask was applied in both groups to restrict the analyses to the same non-resected brain area.

We first characterised surgery-induced changes on white matter and network topology in TLE patients relative to longitudinal measurements in controls over the same time period. Secondly, we investigated the association of those surgery-induced changes with postoperative seizure-freedom. Two complementary approaches were used (Fig. 1).

### 3.1 Surgery-induced changes within white matter fasciculi

**Fig. 2:**
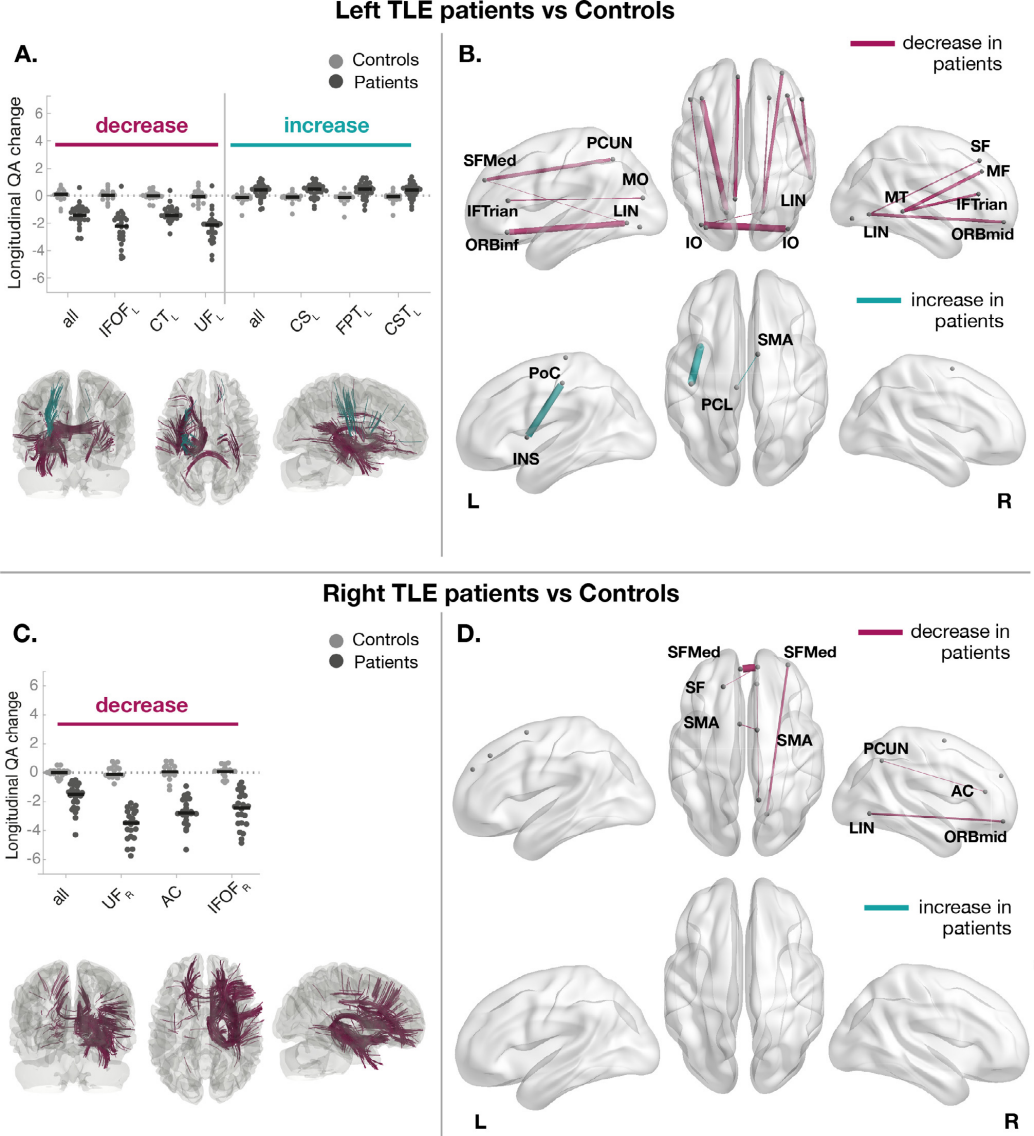
**Significant QA changes between controls and TLE patients, before and after surgery, in parts of tracts revealed by connectometry.** Significant QA changes in subsections of bundles were found between groups (confirmatory grid point: FDR < 0.05; T-score = 3). Subsections of tracts in which patients had a significant QA reduction or increase relative to controls are coloured in purple and green, respectively **(A,C)**. Left and right TLE patients had a greater decrease of QA in ipsilateral inferior fronto-occipital fasciculus (IFOF) and uncinate fasciculus (UF). Greater reductions in QA were also observed in ipsilateral corticothalamic pathway (CT) in left TLE patients and anterior commissure (AC) in right TLE patients. Left TLE also showed a greater increase of QA in ipsilateral corticostriatal pathway (CS), frontopontine tract (FPT) and corticospinal tract (CST). The beeswarm plots show the longitudinal changes in QA between groups for all subsections found by connectometry (label *all* in the plot) and the bundles with greater difference between groups. Each datapoint indicates a single subject. For visualisation purposes, only the three bundles with highest values of cohen-D are presented in the beeswarm plot (see Tables S5 and S6 for complete list). **(B,D)** The tract bundles where subsections were found to be significantly different by connectometry were determined to estimate the AAL regions those subsections are connecting to. The weight of the connections/edges is proportional to the amount of tracts with altered subsections. For visualisation, only the top 70% of strongest connections are shown. All the connections in which the subsections in Fig. 3A and C were found are presented in Figures S3 and S4. A table with the names and abbreviations of all regions and bundles can be found in Tables S7 and S8. AC - *Anterior cingulum*; CUN - *Cuneus*; ORBinf - *Inferior frontal, orbital;* IFTrian - *Inferior frontal, triangular;* IO - *Inferior occipital;* IT - *Inferior temporal;* INS - *Insula;* LIN - *Lingual*; MF - *Middle frontal;* ORBmid - *Middle frontal, orbital;* MO - *Middle occipital;* MT - *Middle temporal;* PCL - *Paracentral lobule;* PoC - *Postcentral;* PCUN - *Precuneus;* SF - *Superior frontal, dorsolateral;* SFMed - *Superior frontal, medial;* SMA - *Supplementary Motor Area.* (For interpretation of the references to colour in this figure legend, the reader is referred to the web version of this article.)

Connectometry detected widespread surgery-induced changes over time in subsections of the tracts located in temporal and extratemporal structures (Fig. 2). Only changes over the first 3–4 months after the surgery were significantly different between patients and controls (FDR < 0.05; T-score = 3; Reduction in QA: cohen-D_Left TLE_ = -2.30, cohen-D_Right TLE_ = -2.76; Increase in QA: cohen-D_Left TLE_ = 0.87). During this period, QA changes in controls were around zero in contrast to TLE patients. Fig. 2A and C show the subsections of white matter tracts with significant changes in QA (FDR < 0.05; T-score = 3) and the three bundles with greater difference in cohen-D between groups. TLE patients showed a greater reduction in QA in the vicinity of the resected tissue, particularly in ipsilateral inferior fronto-occipital fasciculus (IFOF) and uncinate fasciculus (UF). Greater reductions in QA were also observed in ipsilateral corticothalamic pathway (CT) in left TLE patients and anterior commissure (AC) in right TLE patients. The full list of bundles with affected subsections is available in the Supplementary Tables S4-S5. The results are consistent with different t-score thresholds to select local connectomes and at different significance levels (Figure S1 and S2). Figure S3 shows in more detail the location of the subsections in these bundles.

In addition to the reductions in QA, left TLE patients also showed subsections with significant postoperative QA increases over 3–4 months. Specifically, those increases were found in parts of the ipsilateral corticostriatal pathway (CS) and corona radiata, namely in frontopontine tract (FPT) and corticospinal tract (CST) (Fig. 2A).

Fig. 2B and D show the main connections/edges in which the subsections in Fig. 2A and C were found. The main connections/edges with changes in QA were predominantly ipsilateral in right TLE but more bilateral in left TLE. Left and right TLE patients showed greater reduction between ipsilateral lingual (LIN) and orbitofrontal (ORB) cortices. Left TLE also showed a greater decrease in QA in the interhemispheric connections between occipital lobes as compared to right TLE patients, in which the main altered interhemispheric connections were located in frontal cortex. Furthermore, left TLE patients also showed a greater increase in QA in the connections mainly located between ipsilateral insula (INS) and postcentral (PoC) cortices.

Comparable results were obtained with FA (supplemental Figure S4). Greater FA reductions were also observed in the vicinity of the resected tissue, namely in IFOF and inferior longitudinal fasciculus for left TLE and IFOF and UF for right TLE. Similar to QA results, left TLE patients also showed a significantly increased FA in white matter tracts connecting INS and PoC. Right TLE showed increased FA in CS and temporo-pontine tracts, which was not observed using QA. Greater reductions were observed in interhemispheric and contralateral connections in left TLE patients using QA. However, those alterations were less pronounced using FA.

**Fig. 3:**
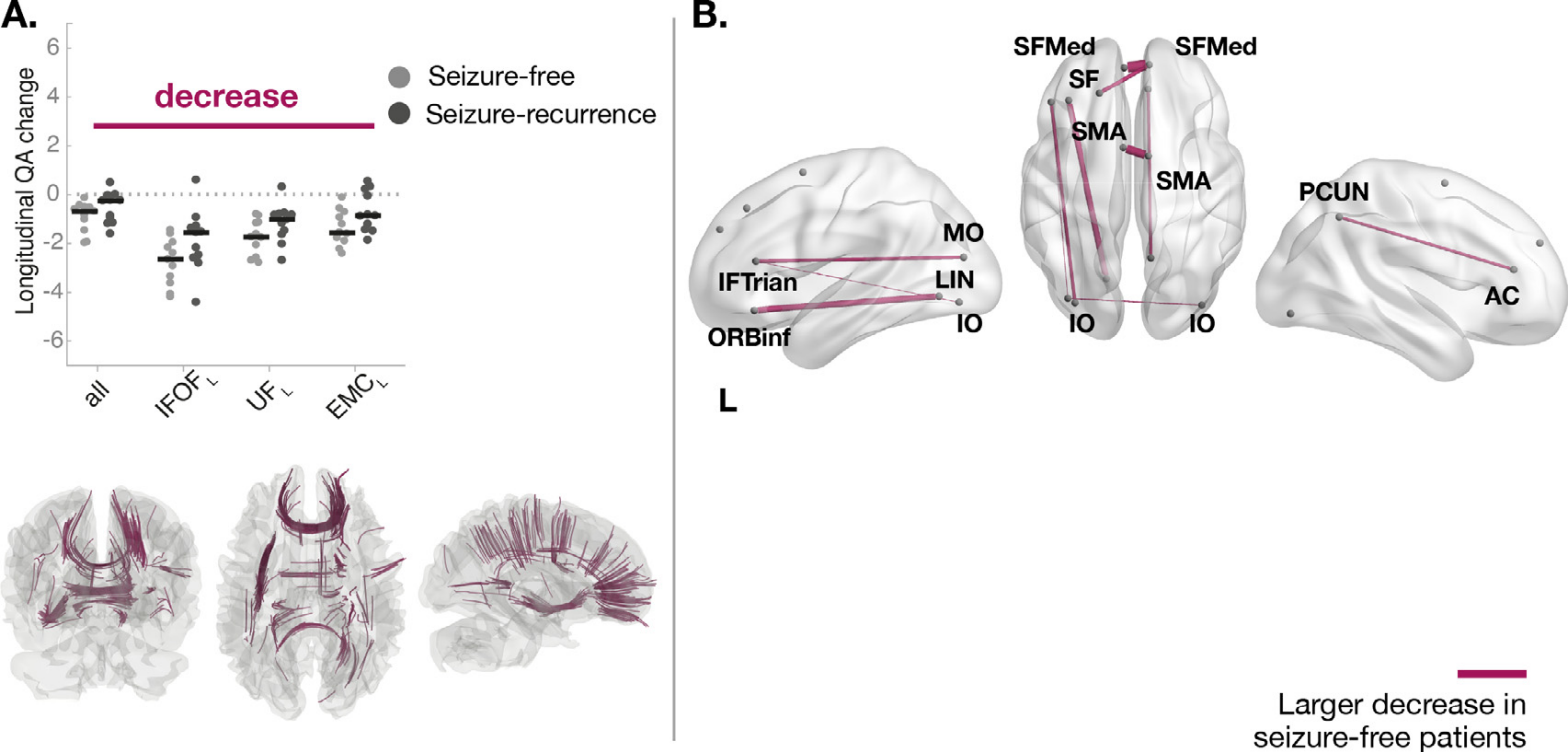
**Tract sections with QA changes over 3 months associated with postoperative seizure freedom in patients with left TLE.** Significant QA changes in subsections of bundles were found between outcome groups (FDR < 0.05; T-score = 3). **(A)** Seizure-free patients had a significantly larger QA reduction over 3–4 months relate to patients with seizure-recurrence in ipsilateral inferior fronto-occipital fasciculus (IFOF), uncinate fasciculus (UF) and extreme capsule (EMC). The beeswarm plot shows the longitudinal changes in QA between groups for all subsections found by connectometry (label *all* in the plot) and the bundles with greater reductions. For visualisation purposes, only the three bundles with the highest Cohen’s d are displayed (Table S7 contains complete list). **(B)** The tract bundles where subsection were found to be sign different by connectometry were determined to estimate the AAL regions those subsections are connecting to. The weight of the connections/edges is proportional to the amount of tracts with altered subsections. For visualisation purposes, a threshold was applied to hide the edges with a weight less than 30% of the strongest connection. A table with the names and abbreviations of all regions and bundles can be found in Tables S7 and S8. AC - *Anterior cingulum*; ORBinf - *Inferior frontal, orbital*; IFTrian - *Inferior frontal, triangular*; IO - *Inferior occipital*; LIN – *Lingual*; MO - *Middle occipital*; PCUN – *Precuneus*; SF - *Superior frontal, dorsolateral*; SFMed - *Superior frontal, medial*; SMA - *Supplementary Motor Area.*

With respect to changes associated with postoperative seizure-freedom, left TLE patients who were seizure free had a widespread decrease in QA greater than patients with seizure-recurrence (FDR < 0.05; T-score = 3, cohen-D = −1.24) (Figs. 3 and S5). As depicted in Fig. 3A, a greater reduction in QA was observed in IFOF, UF and extreme capsule (EMC) (see Supplementary Table S6 for complete list). Fig. 3B depicts the connections where the affected subsections were mainly found, namely between LIN and ORB cortices and inter-hemispheric connections located in the frontal lobe and supplementary motor area (SMA). Figures S6 and S7 show the corresponding results for right TLE patients. Figure S8 shows the corresponding results using FA where greater reductions of FA in seizure-free patients for the tracts connecting contralateral precuneus and anterior cingulum (Figure S8), which is in agreement with our QA findings.

### 3.2 Surgery-induced changes in network topology

**Fig. 4:**
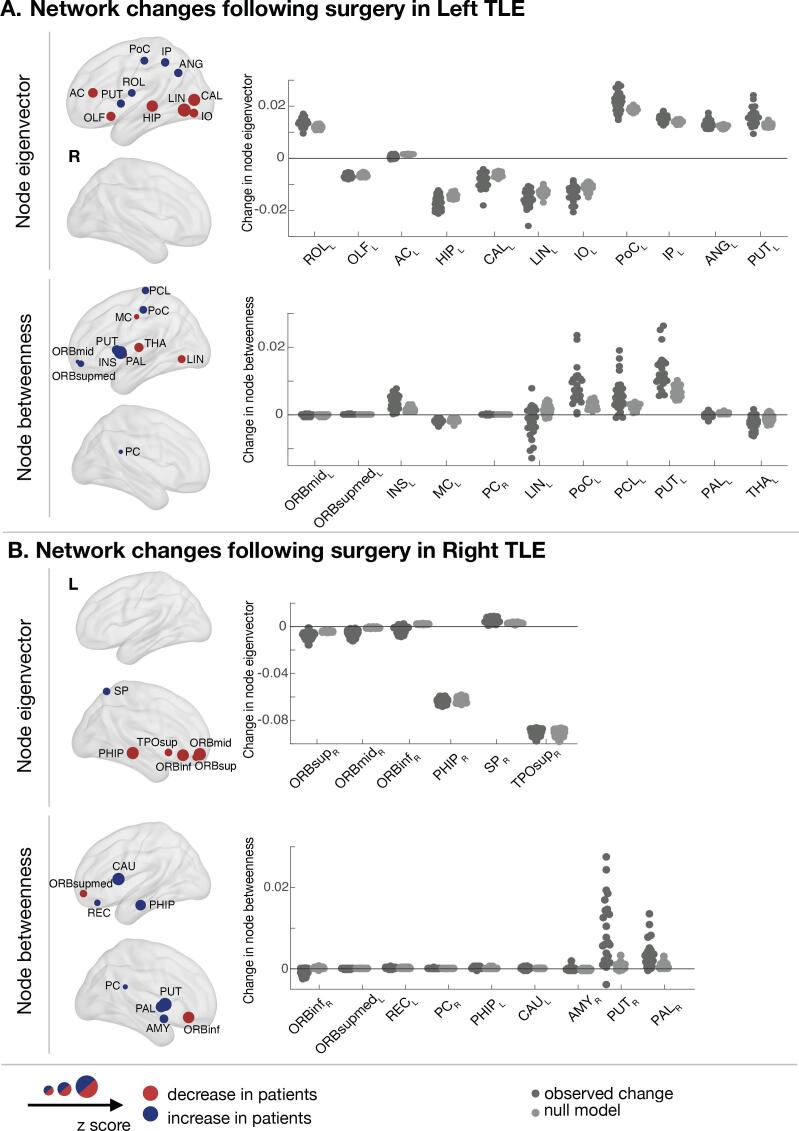
**Changes over 3 months in node eigenvector and node betweenness centrality between patients and controls.** The AAL regions that showed significant differences in node centrality between patients and controls over 3 months are represented by a node/circle and overlaid within a brain volume. Nodes coloured in blue show the AAL regions where patients had an increase in node eigenvector/betweenness centrality in comparison to controls over 3 months. Nodes coloured in red show the AAL regions in which patients had a significant reduction in eigenvector or betweenness centrality in comparison to controls. Scatter plots show the observed and predicted (null model) change in patients after surgery for those AAL regions. Positive values suggest an increase of eigenvector or betweenness centrality following surgery. The scatter plots therefore compare the impact on centrality measures of the surgical resection alone with the overall changes seen post-operatively. A table with the names and abbreviations of all regions can be found in Table S8. AC – *Anterior cingulum*; AMY – *Amygdala;* ANG – *Angular*; CAL – *Calcarine;* CAU – *Caudate;* HIP – *Hippocampus;* INS – *Insula*; IO – *Inferior occipital;* IP – *Inferior parietal*; LIN – *Lingual;* MC - *Middle cingulum;* OLF – *Olfactory*; ORBinf - *Inferior frontal, orbital*; ORBmid - *Middle frontal, orbital*; ORBsup - *Superior frontal, orbital*; ORBsupmed - *Superior frontal, medial*; PAL – *Pallidum*; PC - *Posterior cingulum;* PCL – *Paracentral lobule*; PHIP – *Parahippocampal*; PoC – *Postcentral*; PUT – *Putamen;* REC – Rectus; ROL *- Rolandic operculum*; SP – *Superior parietal*; THAL – *Thalamus*; TPOsup – *Superior Temporal Pole*. (For interpretation of the references to colour in this figure legend, the reader is referred to the web version of this article.)

Graph-theory identified surgery-induced changes to graph theory metrics between patients and controls mainly in the ipsilateral side. In the left panels of Fig. 4a we show those regions with altered metrics in patients relative to controls. Note that voxels within the resection area are excluded for all subjects (including controls) in this analysis to therefore allow visualisation of only the most pronounced changes outside of the resection. Over the first 3–4 months following surgery, only local changes in strength, clustering coefficient, eigenvector and betweenness centrality were significantly different between groups (FDR p-value < 0.005).

To support the interpretation of these results, scatter plots in Fig. 4 show the observed and predicted changes in patients by the observed and null model, respectively. Although widespread reductions and increases of network metrics were identified by the null and observed model, we only plotted the changes for a set of network metrics, which showed a significant longitudinal difference between patients and controls over 3–4 months following surgery (i.e. corresponding to those regions in the left panels).

**Fig. 5:**
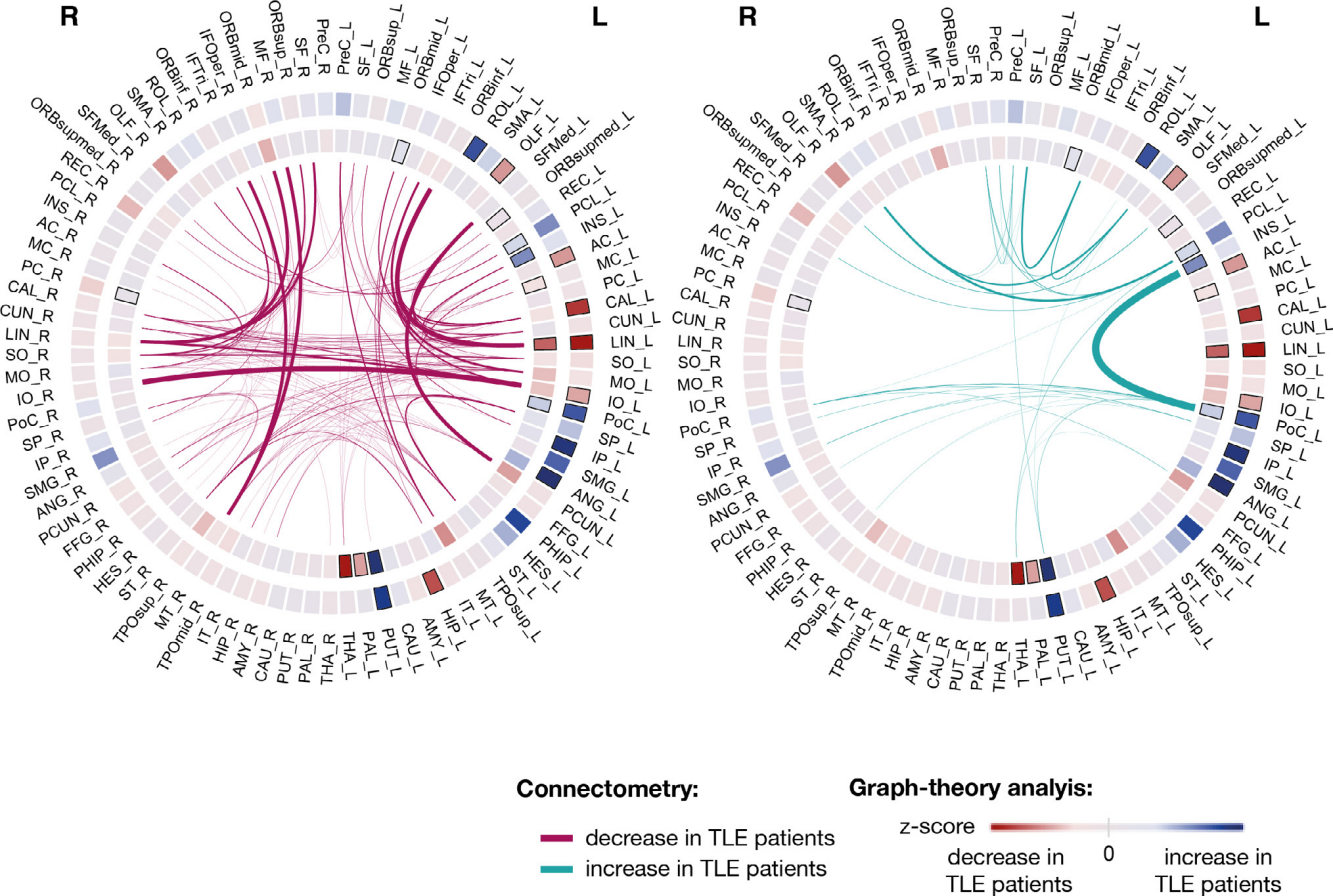
**Connectogram comprising the surgery induced changes detected by graph-theory and connectometry in left TLE patients.** Graph theory: Graph-theory changes between patients and controls are displayed in the circles. The outer and inner circle indicates the z-score between groups for eigenvector and betweenness centrality, respectively. Z-score values are represented by a red-blue gradient, in which red indicates the minimum calculated z-score and blue the maximum value. Z-score for node eigenvector changed between −2.35 and 1.17 and z-score for node betweenness between −1.83 and 3.15. The black rectangles indicate the AAL regions with significant change in node eigenvector or node betweenness centrality after FDR correction. Connectometry: The connections with significant changes in their subsections as identified by connectometry are displayed in the centre. The weight of the connection is proportional to the amount of streamlines in which QA changes were detected. A table of AAL region names and abbreviations can be found in Table S8. (For interpretation of the references to colour in this figure legend, the reader is referred to the web version of this article.)

**Fig. 6:**
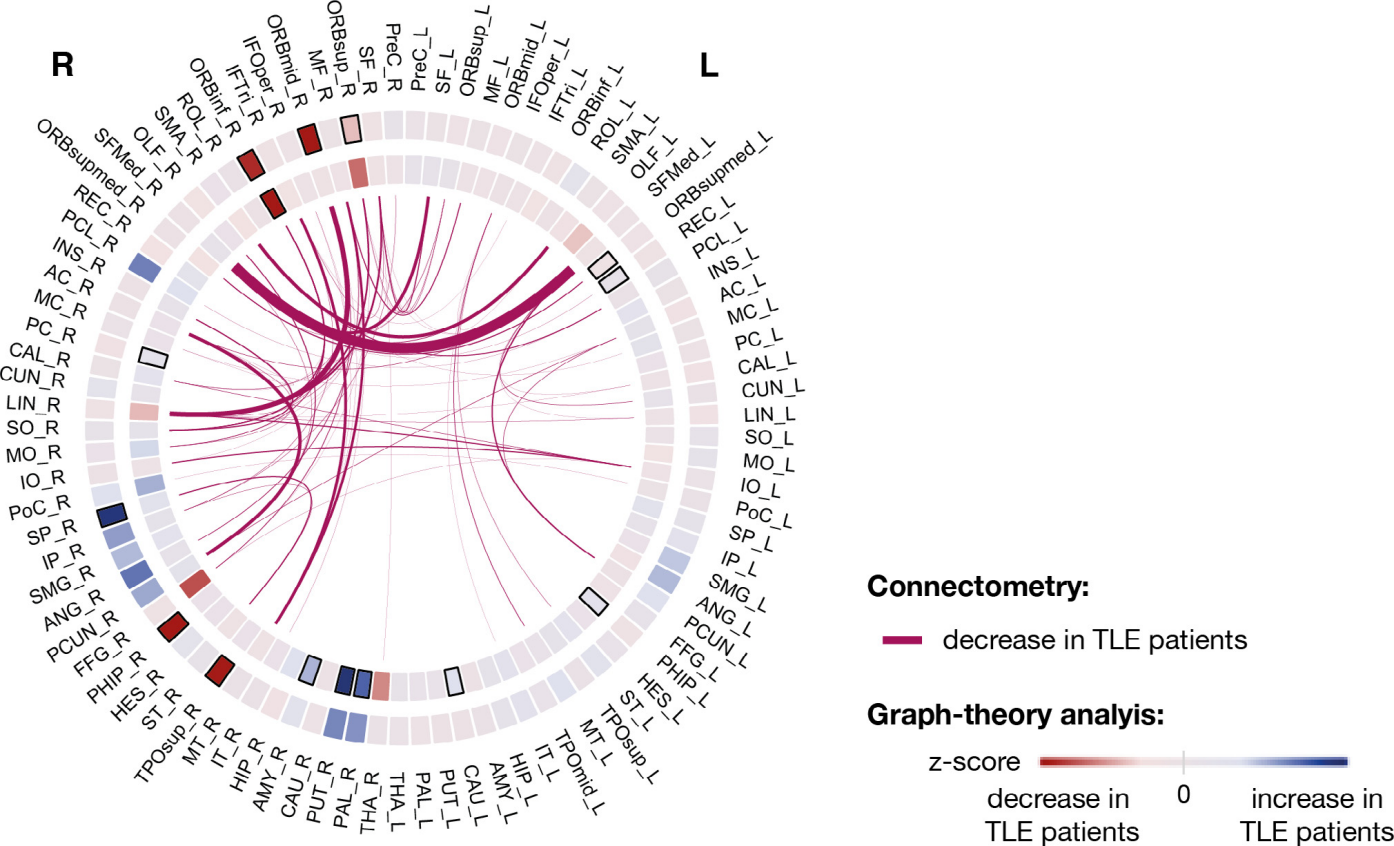
**Connectogram comprising the surgery induced changes detected by graph-theory and connectometry in right TLE patients.** Graph theory: Graph-theory changes between patients and controls are displayed in the circles. The outer and inner circle indicates the z-score between groups for eigenvector and betweenness centrality, respectively. Z-score values are represented by a red-blue gradient, in which red indicates the minimum calculated z-score and blue the maximum value. Z-score for node eigenvector changes between −4.13 and 1.89 and z-score for node betweenness between −2.02 and 2.50. The black rectangles indicate the AAL regions with significant change in node eigenvector or node betweenness centrality after FDR correction. Connectometry: The connections with significant changes in their subsections as identified by connectometry are displayed in the centre. The weight of the connection is proportional to the amount of streamlines in which QA changes were detected. A table of AAL region names and abbreviations can be found in Table S8. (For interpretation of the references to colour in this figure legend, the reader is referred to the web version of this article.)

Node eigenvector centrality measures the influence a region has on a network. When removing a region in full or partially, nodes connected to that region or with neighbours connected to it might lose influence on the network. Longitudinal changes in node eigenvector centrality were ipsilateral for both left and right TLE. In left TLE, a decrease in node eigenvector centrality in patients in comparison to controls was observed in hippocampus, olfactory, anterior cingulum and occipital lobe, namely in LIN, calcarine (CAL) and inferior occipital (IO). These changes were expected by the null model and a significant decrease in QA in the white matter tracts connected to LIN, CAL, IO and anterior cingulum was also observed using connectometry (Fig. 5). Therefore, these regions and/or their neighbours were likely to be connected to the resected tissue or having connections passing through it. An increase of eigenvector centrality was also observed in putamen (PUT), rolandic operculum, and parietal lobe. Similarly, these changes were expected by the null model. However, PoC and PUT showed a much higher increase postoperatively than the one predicted by the null model, suggesting a possible reorganisation to reinforce these regions to pass shortest topological paths. This is supported by connectometry results, in which PoC and PUT tracts showed a significant QA increase over time (Fig. 6). In right TLE, a decrease of node eigenvector centrality, node clustering coefficient and strength were observed in ORB cortex and temporal lobe (Fig. 4 and Figure S9). Those changes were expected by the null model and some of these regions showed a significant QA reduction in their connections (Fig. 6). Similarly to left TLE, these regions and/or their neighbours were likely to have connections passing or ending in the resected area.

Node betweenness centrality measures how important a region is by virtue of being on shortest paths between other regions: i.e. in integrating information across the network. When removing a region in full or partially, the shortest path between regions can change by traversing other areas. Thus, the betweenness centrality in this region can either increase or decrease following surgery (Taylor et al., 2018). As depicted in Fig. 4, changes in node betweenness centrality were mainly ipsilateral and more widely distributed in left TLE patients, while in right TLE were bilateral and mainly in central structures. A decrease of node betweenness were observed for ipsilateral thalamus, middle cingulum in left TLE and ORB cortex in right TLE. The decrease of betweenness centrality in these regions is expected by the null model Fig. 4 (right panel). A significant QA reduction of these regions’ connections is also shown by connectometry in Fig. 6. Therefore, these regions were likely to be connected to the resected tissue or having connections passing through it. In addition, LIN also showed decreased node betweenness centrality in left TLE in comparison to controls. Although by the null model it was expected an increase of betweenness centrality, it was observed a reduction by the observed model (Fig. 4, right panel). Connectometry also showed a significant QA reduction of connections between LIN to/from ORB cortex. This suggests secondary degeneration of LIN connections not assessed by the null model or brain reorganization. With disconnection of some tracts following surgery, the shortest paths redirect and might also lead to increase of node betweenness centrality. Left TLE patients showed increase of node betweenness in ipsilateral ORB cortex, PoC, INS, paracentral lobule, PUT, pallidum (PAL) and contralateral posterior cingulum (PC). In contrast, right TLE patients showed increased node betweenness centrality in parahippocampal, gyrus rectus, PC and several central structures such as PUT, PAL, caudate and amygdala. Most of the regions showing an increase of betweenness centrality in left TLE also showed significantly increased QA in their white matter tracts to other regions, namely ipsilateral PoC, PCL, INS and PUT. In the observed change model those regions showed an increase of betweenness centrality greater than expected, suggesting possible reinforcement of those regions to be involved in the shortest paths. In right TLE, the observed model also showed an increase of betweenness centrality much higher than the null model for ipsilateral PAL and PUT. Although a significant increase of QA in their connections was not observed using connectometry, it is likely that other white matter alterations occurred in neighbouring nodes leading to a reinforcement of their role on passing shortest paths.

No longitudinal changes in network topology after the surgery were found to be significantly different between TLE patients with different postoperative outcomes of seizure-freedom.

## 4 Discussion

In this study we performed a comprehensive investigation of longitudinal white matter QA changes after epilepsy surgery and their association with postoperative seizure freedom. In the 3–4 months after surgery, we found surgery-induced changes in white matter and network topology in the temporal lobe and beyond.

First, surgery induced longitudinal white matter changes were primarily ipsilateral in right TLE but more bilateral in left TLE. The largest post-operative reductions in QA, likely related to primary Wallerian degeneration, were in tracts which were transected by surgery (ipsilateral UF and IFOF). Decreased node eigenvector and betweenness centrality was therefore expected and observed in regions partially resected and/or connected to the resected tissue. Secondary processes may also explain further reductions in QA, namely in white matter tracts connecting lingual and orbitofrontal cortices.

Secondly, left TLE patients showed increased QA in cortico-striatal and corona radiata tracts, likely related to improvements in verbal fluency (Yogarajah et al. (2010). These tracts mainly connect the postcentral gyrus and insula, which both showed a higher increase of betweenness centrality after surgery than expected by the null model, suggesting possible strengthening of these connections.

Thirdly, with respect to postoperative seizure outcome, left TLE seizure-free patients showed greater QA reductions compared to seizure-recurrence patients in interhemispheric connections in frontal lobe and tracts transected by surgery (ipsilateral UF and IFOF). Seizure-freedom was not significantly related to alterations in the network topology or resection volume.

### 4.1 Surgery-related effect on white matter

More bilateral and widespread white matter disruptions have been reported in patients with left TLE as opposed to right TLE using preoperative data (Ahmadi et al., 2009, Kemmotsu et al., 2011, Besson et al., 2014). Following the surgery, left TLE patients also had more bilateral disruptions in white matter than right TLE 3–4 months after surgery (Fig. 5, Fig. 6), suggesting different mechanisms of degeneration.

Our observation of reduced QA in tracts close to the resection area agrees with previous literature. Specifically, postoperative reductions in UF and IFOF have been described by McDonald et al., 2010, Yogarajah et al., 2010, Winston et al., 2014. As in our study, Yogarajah et al. (2010) also reported a decrease in AC following surgery in right TLE patients, which may be explained by larger right-sided resections compared to smaller left-sided resections. Furthermore, reduced QA might have also been explained by secondary degeneration. An increase of betweenness centrality was expected in the lingual cortex as shortest paths were expected to be rerouted to this node by the null model. However, in postoperative data we observed a decrease of betweenness centrality, supported by a reduction of QA in its connections to orbitofrontal cortex. Atrophy propagation through white matter or reorganisation to reroute shortest paths due to the loss of function of lingual cortex and neighbouring regions following surgery, could explain a secondary degeneration in this region. Reduced QA, a measure of fibre volume fraction and highly correlated with FA, can be caused by Wallerian degeneration as surgery disconnects white matter tracts, potentially causing myelin degradation and reduction in axonal packing (Concha et al., 2007). We build on this by demonstrating that there are substantial changes beyond normative changes over the same time period. The results are also spatially similar to those obtained with FA. A reduction of FA suggested axonal damage or Wallerian degeneration and demyelination 3 months following the surgery.

In addition to previously described QA reductions, we found QA increases in corticopontine tracts, CS and CST. Winston et al. (2014) reported a significant increase in FA following ATLR in the corona radiata, which includes tracts from CST and corticopontine bundles. Following a mean of 4.5 months after ATLR, Yogarajah et al. (2010) also reported a significant increase in FA in CST and corticopontine tracts associated with increased verbal fluency, suggesting it was related to early structural plasticity of language networks. Furthermore, the affected subsections found in these bundles involved insula, a network hub for perception of speech and receptive and expressive language (Oh et al., 2014). Insula showed a significant increase in betweenness centrality following surgery as expected by the null model. However, in our postoperative data we saw an even higher increase than expected, suggesting reinforcement of the redistribution of shortest paths through this hub for language recovery (Fig. 4). As connections to/from the posterior part of the insula might be resected in ATLR (Taylor et al., 2018), future research should examine if larger resections of insular connections lead to worse language recovery after surgery. In agreement with our findings, McDonald et al. (2010) also reported an increase in CS, although in their study of 9 patients it did not meet significance p = 0.079. CS pathways are related with the limbic system (Shipp, 2017), thus an increase in QA might be related with structural plasticity. Connectometry results showing an increase in FA were spatially similar to those using QA, namely in CS and corticopontine bundles. An increase of FA might be due to several mechanisms including increased myelination, axon diameter, fiber density or changes in axonal membrane structure (Moore et al., 2017).

Since connectometry isolates only the affected segment of the tract, it achieves greater statistical sensitivity. In contrast, graph-theory may only detect significant changes in a node/region when there is a considerable amount of nearby affected connections (Yeh et al., 2018). Fig. 5, Fig. 6 integrate the results from graph theory and connectometry in terms of the AAL regions involved in white matter longitudinal changes. AAL regions showing changes in network topology are consistent with the AAL regions connected by tracts with greater affected subsections. However, connectometry also detected affected subsections in tracts connecting other regions. Taylor et al., (2018) inferred the impact of surgical resection on network topology using preoperative data as in the null model in this study. Similarly to our study, Taylor et al., (2018) reported widely distributed increases in betweenness centrality, suggesting rerouting of shortest paths and strengthening of tracts to avoid cognitive deficits. Comparing null model with the postoperative data, we observed that rerouting of shortest paths occurs similarly to the null model. However, strengthening of some white matter tracts leads to a reinforcement of the rerouting for some regions, namely in putamen in left and right TLE and insula, postcentral gyrus and paracentral lobule in left TLE. A weakening of the expected white matter tracts to reroute was also observed either due to atrophy propagation or reorganization.

In terms of timescale of the imaging, our analysis is performed during the chronic phase several months after surgery as were most others (McDonald et al., 2010, Li et al., 2019, Winston et al., 2014). Our findings of no substantial QA changes between 3 and 12 months is in agreement with previous studies (McDonald et al., 2010), suggesting the largest changes occur within the first months after surgery. However, the lower sample size of 12 month imaging may also contribute to the lack of significant results during this time.

### 4.2 White matter alterations associated with post-surgery seizure relapse

We observed postoperative QA decreases 3–4 months after surgery associated with postoperative seizure freedom, which has not been shown previously to our knowledge. We found that greater chances of postoperative seizure freedom relate to greater QA reductions in interhemispheric connections and in the vicinity of resection area (Fig. 3). The greater reductions in seizure free patients may be explained by larger resection volume (Shamim et al., 2009). However, in our cohort the resection masks were consistent across patients (Supplemental Results: Consistency of resection masks) and no significant differences were observed in total resection volume between patients with different outcomes. When regressing out the resection volume, connectometry results still show greater reductions in seizure free patients in these bundles (Supplemental Figure S10). Nevertheless, small variations in the extent of resection, especially in piriform cortex, might have driven larger reductions in QA. Galovic et al., (2019) recently reported that if surgery included the piriform cortex, there was a greater chance of seizure freedom. In right TLE, we found widespread differences between outcome groups in subsections of tracts. However, given the small sample size for right TLE outcomes we caution against overinterpretation of this result.

With respect to the network topology, the affected subsections of tracts significantly different between groups did not contribute to changes in the network topology. Preoperatively, seizure-freedom has been associated with hub redistributions and reduced local efficiency, which might reduce the spread of epileptic discharges (Ofer et al., 2019). Taylor et al., (2018) inferred the impact of surgical resection on network topology and reported a reduction in local efficiency. Similar to the present study, reductions in node local efficiency were not associated with postoperative seizure-freedom. Using machine learning, Taylor et al., 2018 reported that changes in the number of tracts between some regions in frontal and temporal lobes were predictive of seizure freedom. Similarly, we observed a reduction in QA in connections in the temporal and frontal lobes using connectometry. Furthermore, we also report greater reductions in inter-hemispheric connections in frontal and occipital lobe. Inferring the impact of surgical resection in preoperative data, as in Taylor et al., (2018), does not allow to take into account secondary degeneration or plasticity. This might explain why in our longitudinal study we also report greater reductions in inter-hemispheric connections.

### 4.3 Limitations

Our study has some limitations that should be considered. First, we only analysed *changes* over time which were made possible by the longitudinal aspect of our data. We did not compare our findings to cross sectional pre- or post-operative diffusion MRI data which may, in isolation, explain postoperative seizure-freedom (Keller et al., 2017, Bonilha et al., 2013, Bonilha et al., 2015, Bonilha and Keller, 2015). Future studies could investigate pre- and postoperative networks, in addition to the changes in those networks as described here. Second, our sample size - although fairly large compared to other similar studies of white matter postoperative changes - is small for our analysis of post-surgical patient outcomes (group size: Left TLE − 11 seizure-free and 11 seizure-recurrence; Right TLE − 5 seizure-free and 12 seizure-recurrence). A way to boost sample size is to perform an ipsi- and contralateral analysis as in some previous studies (Seidenberg et al., 2005). However, given that right-sided resections are substantially larger than left sided-resections we chose not to do that here - further supported by the different findings ipsilateral to left and right TLE. Third, our sample size for 12 month scans is limited to only 13 patients.

## 5 Conclusion

Our results confirm previous findings of widespread alterations after epilepsy surgery beyond the site of resection. These changes include primary degeneration proximal to the resection, and potentially secondary adaptive changes in more distant areas. Using a null model of surgery we showed these changes to be above and beyond those expected through the removal of connections alone. Greater reductions in QA were related to postoperative seizure freedom in left TLE suggesting a greater impact on local tract properties. Our work contributes to an improved understanding of how brain networks change in response to surgery.

## CRediT authorship contribution statement

**Nádia Moreira da Silva:** Conceptualization, Methodology, Software, Investigation, Writing - original draft, Visualization. **Rob Forsyth:** Supervision, Methodology, Writing - review & editing. **Andrew McEvoy:** Writing - review & editing. **Anna Miserocchi:** Writing - review & editing. **Jane de Tisi:** Writing - review & editing, Data curation. **Sjoerd B. Vos:** Writing - review & editing, Data curation, Resources. **Gavin P. Winston:** Writing - review & editing, Data curation, Resources, Funding acquisition. **John Duncan:** Writing - review & editing, Data curation, Resources, Funding acquisition. **Yujiang Wang:** Writing - review & editing, Resources. **Peter N. Taylor:** Conceptualization, Supervision, Writing - review & editing, Data curation, Resources, Validation, Funding acquisition.

## Declaration of Competing Interest

The authors declare that they have no known competing financial interests or personal relationships that could have appeared to influence the work reported in this paper.
