# Supplementary material for: Network reorganisation following anterior temporal lobe resection and relation with post-surgery seizure relapse: A longitudinal study
Source: Neuroimage Clin. 2020 Jun 26;27:102320. doi: 10.1016/j.nicl.2020.102320 (PMC7334605; doi:10.1016/j.nicl.2020.102320)
Supplement: Supplementary data 1 [file mmc1.docx]

# **Supplementary Data**

## **Supplementary Tables**

**Table S1.** Demographic and clinical data of patients and controls with imaging available 12 months after surgery.

|  | Left TLE | Right TLE | Controls | Significance |
| --- | --- | --- | --- | --- |
| *Subjects (n)* | 8 | 5 | 17 | - |
| *Sex*  *(Male/Female)* | 3/5 | 0/5 | 8/6 | Chi2 _(Left TLE; Controls)_ = 0.786  p _(Left TLE; Controls)_ = 0.375  Chi2 _(Right TLE; Controls)_ = 4.935  **p _(Right TLE; Controls)_ = 0.026** |
| *Age at baseline scan* | 38.43±11.56 | 35.60±11.56 | 38.14±10.09 | t _(Left TLE; Controls)_ = 0.060  p _(Left TLE; Controls)_ = 0.953  t _(Right TLE; Controls)_ = -0.466  p _(Right TLE; Controls)_ = 0.647 |
| *Onset* | 15.38±9.41 | 13.20±3.56 | - | - |
| *Age at surgery* | 38.68±11.49 | 36.72±11.61 | - | - |
| *Epilepsy Duration* | 23.30±17.19 | 23.52±10.88 | - | - |
| *Status Epilepticus (n)* | 2 | 0 | - | - |
| *Secondary generalised seizures (n)* | 7 | 3 | - | - |
| *Hippocampal Sclerosis (n)* | 6 | 3 | - | - |
| *Seizure-Free for 5 years (n)* | 4 | 0 | - | - |
| *Seizure recurrence (n)* | 3 | 3 | - | - |

**Table S2.** Complete preoperative clinical information of all patients with postoperative ILAE seizure outcomes and relapse data

| IDP | Sex | Side | Onset | Surg | Dur. | Bas. | SE | SGS | HS | ILAE Y1 | ILAE Y2 | ILAE Y3 | ILAE Y4 | ILAE Y5 | SF | Rel. |
| --- | --- | --- | --- | --- | --- | --- | --- | --- | --- | --- | --- | --- | --- | --- | --- | --- |
| 1 | F | L | 2.0 | 19.1 | 17.1 | 18.5 | N | Y | Y | 1 | 2 | 3 | 1 | 5 | N | Y |
| 2 | F | L | 13.0 | 20.3 | 7.3 | 19.8 | Y | Y | Y | 1 | 1 | 1 | 1 | - | N | N |
| 3 | F | L | 0.9 | 27.3 | 26.4 | 26.8 | N | Y | N | 1 | 1 | 1 | 1 | 1 | Y | N |
| 4 | M | L | 16.0 | 52.9 | 36.9 | 51.3 | N | Y | Y | 1 | 1 | 1 | 1 | 1 | Y | N |
| 5 | M | L | 32.0 | 40.6 | 8.6 | 40.6 | N | N | N | 1 | 1 | 1 | 1 | - | N | N |
| 6 | F | L | 34.0 | 38.6 | 4.6 | 37.9 | N | Y | Y | 2 | 2 | 2 | 2 | 1 | N | Y |
| 7 | M | L | 22.0 | 33.9 | 11.9 | 32.6 | N | Y | N | 1 | 1 | 4 | 1 | - | N | Y |
| 8 | M | L | 2.0 | 39.1 | 37.1 | 38.2 | N | Y | Y | 1 | 1 | 1 | 1 | 1 | Y | N |
| 9 | M | L | 8.0 | 40.3 | 32.3 | 38.9 | N | Y | Y | 2 | 1 | 1 | 1 | - | N | Y |
| 10 | F | L | 17.0 | 29.3 | 12.3 | 27.4 | N | Y | Y | 1 | 1 | - | - | - | N | N |
| 11 | F | L | 21.0 | 42.3 | 21.3 | 40.9 | N | N | Y | 4 | 4 | 4 | 4 | 1 | N | Y |
| 12 | F | L | 38.0 | 43.2 | 5.2 | 43.1 | N | N | N | 1 | 1 | 1 | 1 | 1 | Y | N |
| 13 | M | L | 31.0 | 46.0 | 15.0 | 45.9 | N | Y | Y | 1 | 2 | 3 | 3 | 1 | N | Y |
| 14 | F | L | 3.0 | 49.1 | 46.1 | 48.8 | N | Y | Y | 1 | 1 | 1 | 1 | 1 | Y | N |
| 15 | M | L | 23.0 | 28.6 | 5.6 | 28.2 | Y | Y | N | 1 | 1 | 1 | 1 | 1 | Y | N |
| 16 | F | L | 7.0 | 46.0 | 39.0 | 45.8 | N | Y | Y | 1 | 1 | 1 | 1 | 1 | Y | N |
| 17 | M | L | 12.0 | 45.1 | 33.1 | 44.9 | N | Y | Y | 1 | 1 | 1 | 1 | 1 | Y | N |
| 18 | F | L | 11.0 | 47.7 | 36.7 | 47.6 | N | Y | Y | 5 | 5 | 5 | 5 | 5 | N | Y |
| 19 | F | L | 16.0 | 31.0 | 15.0 | 30.8 | Y | Y | Y | 1 | 1 | 1 | 1 | 1 | Y | N |
| 20 | M | L | 7.0 | 26.4 | 19.4 | 26.3 | N | N | Y | 1 | 1 | 1 | - | - | N | N |
| 21 | F | L | 23.0 | 26.6 | 3.6 | 26.5 | N | N | N | 2 | 4 | 2 | 2 | 2 | N | Y |
| 22 | M | L | 17.0 | 32.5 | 15.5 | 32.1 | N | N | Y | 1 | 1 | 1 | 1 | 1 | Y | N |
| 23 | M | L | 1.0 | 47.3 | 46.3 | 46.2 | N | Y | Y | 1 | 1 | 1 | 1 | 1 | Y | N |
| 24 | M | L | 20.0 | 35.2 | 15.2 | 33.7 | N | Y | N | 1 | 1 | 4 | 3 | 3 | N | Y |
| 25 | F | L | 31.0 | 39.9 | 8.9 | 38.9 | N | Y | N | 1 | 2 | 1 | 1 | 1 | N | Y |
| 26 | M | L | 13.0 | 21.1 | 8.1 | 20.8 | N | Y | Y | 1 | 1 | 1 | 1 | 3 | N | Y |
| 27 | F | R | 14.0 | 22.2 | 8.2 | 21.1 | N | Y | Y | 2 | 3 | 4 | 4 | 4 | N | Y |
| 28 | M | R | 31.0 | 42.6 | 11.6 | 41.6 | - | - | Y | 1 | 1 | 1 | 1 | 1 | Y | N |
| 29 | F | R | 15.0 | 20.2 | 5.2 | 19.9 | N | Y | N | 1 | 1 | 1 | 2 | 4 | N | Y |
| 30 | F | R | 10.0 | 41.9 | 31.9 | 40.5 | - | - | N | 1 | 1 | 1 | 1 | - | N | N |
| 31 | F | R | 17.0 | 39.2 | 22.2 | 38.2 | N | Y | Y | 1 | 1 | 1 | - | - | N | N |
| 32 | F | R | 23.0 | 44.7 | 21.7 | 42.3 | N | N | N | 2 | 1 | 1 | 4 | - | N | Y |
| 33 | F | R | 7.0 | 48.1 | 41.1 | 46.7 | N | Y | Y | 1 | 1 | 1 | - | - | N | N |
| 34 | F | R | 50.0 | 68.2 | 18.2 | 66.8 | Y | Y | N | 4 | 4 | 4 | 4 | 4 | N | Y |
| 35 | M | R | 14.0 | 47.7 | 33.7 | 44.9 | N | N | N | 4 | 4 | 2 | 2 | 2 | N | Y |
| 36 | F | R | 22.0 | 48.0 | 26.0 | 46.8 | N | Y | Y | 1 | 1 | 1 | 1 | 1 | Y | N |
| 37 | F | R | 1.5 | 31.7 | 30.2 | 30.3 | N | N | Y | 1 | 1 | 1 | 1 | 1 | Y | N |
| 38 | F | R | 10.0 | 32.4 | 22.4 | 31.2 | N | N | N | 1 | 1 | - | - | - | N | N |
| 39 | F | R | 7.0 | 25.3 | 18.3 | 24.8 | N | Y | N | 1 | 1 | 1 | 1 | 1 | Y | N |
| 40 | F | R | 3.0 | 54.5 | 51.5 | 53.2 | N | Y | Y | 2 | 3 | 3 | - | - | N | Y |
| 41 | F | R | 18.0 | 47.0 | 29.0 | 45.3 | N | Y | N | 4 | 4 | 4 | - | - | N | Y |
| 42 | F | R | 12.0 | 48.2 | 36.2 | 47.3 | N | Y | Y | 1 | 1 | 1 | - | - | N | N |
| 43 | F | R | 15.0 | 23.4 | 8.4 | 22.6 | N | Y | N | 1 | 1 | 1 | 3 | 3 | N | Y |
| 44 | F | R | 23.0 | 36.6 | 13.6 | 35.7 | N | N | N | 2 | 1 | 2 | 2 | 1 | N | Y |
| 45 | F | R | 9.0 | 42.5 | 33.5 | 41.9 | N | Y | Y | 1 | 1 | 1 | 1 | 1 | Y | N |
| 46 | F | R | 2.0 | 57.3 | 55.3 | 57.0 | N | Y | Y | 1 | 1 | 1 | 3 | 1 | N | Y |
| 47 | F | R | 16.0 | 51.8 | 35.8 | 50.9 | N | Y | Y | 3 | 3 | 2 | 2 | 2 | N | Y |
| 48 | F | R | 9.0 | 28.5 | 19.5 | 27.3 | N | N | N | 2 | 1 | 4 | 4 | 4 | N | Y |

**Abbreviations:** *Sex:* male (M)/female (F); ); *Side*: side of surgery left (L) or right (R); *Onset:* epilepsy onset age in years; *Surg.:* age at surgery in years; *Dur.:* epilepsy duration in years; *Bas*.: age at baseline scan; *SE:* history of status epilepticus in yes (Y) or no (N); *SGS:* evidence of secondary generalised seizures in yes (Y) or no (N); *ILAE Y1, Y2, Y3, Y4,Y5:* post-surgery seizure outcome at year 1, 2, 3, 4 and 5; *SF: seizure-free for 5 years in yes (Y) or no (N); Rel.: seizure-recurrence over 5 years in yes (Y) or no (N).*

**Table S3.** List of network metrics used to quantify network segregation, integration and centrality.

| Properties | Metrics |
| --- | --- |
| Segregation | Average Clustering Coefficient |
|  | Local Clustering Coefficient |
|  | Local Efficiency |
|  | Small-worldness |
| Integration | Characteristic Path Length |
|  | Global Efficiency |
|  | Small-worldness |
| Centrality | Closeness |
|  | Betweenness |
|  | Eigenvector |
|  | Strength |

**Table S4.** **White matter changes significantly different between left TLE patients and controls 3 months after surgery.** The subsections were grouped in the set of bundles of the connectome atlas. Cohen’s d between groups, number of altered streamlines (No streamlines) and mean length for each bundle is presented below. The Cohen’s d between groups taking into account all the subsections found by connectometry without any grouping is also presented with the label “all bundles”.

| Bundles | No streamlines | Mean streamline length (± standard deviation) | Cohen’s d | QA Finding |
| --- | --- | --- | --- | --- |
| AC | 174 | 30.44 ± 8.51 | -2.24 | Reduction |
| AF_L | 38 | 23.37 ± 2.56 | -1.72 | Reduction |
| AF_R | 4 | 20.50 ± 0.87 | -0.27 | Reduction |
| AR_L | 2 | 21.00 ± 1.00 | -0.78 | Reduction |
| AR_R | 2 | 21.00 ± 1.00 | -0.50 | Reduction |
| C_L | 25 | 27.52 ± 7.00 | -1.05 | Reduction |
| C_R | 3 | 24.00 ± 5.66 | -0.97 | Reduction |
| CC | 389 | 30.14 ± 9.33 | -0.91 | Reduction |
| CS_L | 19 | 23.68 ± 3.57 | -1.72 | Reduction |
| CS_R | 15 | 24.00 ± 2.42 | -0.46 | Reduction |
| CST_L | 57 | 25.61 ± 4.38 | -1.46 | Reduction |
| CST_R | 91 | 30.73 ± 8.81 | -0.46 | Reduction |
| CT_L | 81 | 27.70 ± 6.89 | -2.87 | Reduction |
| CT_R | 15 | 26.00 ± 5.16 | -0.45 | Reduction |
| EMC_L | 5 | 24.80 ± 5.00 | -2.19 | Reduction |
| F_L | 82 | 32.41 ± 10.95 | -2.45 | Reduction |
| F_R | 5 | 22.00 ± 1.26 | -1.37 | Reduction |
| FPT_L | 1 | 24.00 ± 0 | -0.38 | Reduction |
| FPT_R | 59 | 29.66 ± 8.52 | -0.50 | Reduction |
| IFOF_L | 667 | 41.31 ± 9.93 | -2.94 | Reduction |
| IFOF_R | 36 | 25.44 ± 5.33 | -0.44 | Reduction |
| ILF_L | 65 | 25.11 ± 4.24 | -2.24 | Reduction |
| ILF_R | 2 | 21.00 ± 1.00 | -0.37 | Reduction |
| MdLF_L | 27 | 23.48 ± 2.41 | -1.87 | Reduction |
| MdLF_R | 3 | 24.67 ± 0.94 | -0.50 | Reduction |
| OPT_L | 5 | 23.60 ± 1.50 | -1.71 | Reduction |
| OPT_R | 23 | 25.30 ± 3.76 | -0.38 | Reduction |
| OR_L | 4 | 22.00 ± 1.41 | -0.94 | Reduction |
| OR_R | 1 | 22.00 ± 0 | -0.26 | Reduction |
| PPT_L | 90 | 25.56 ± 3.69 | -1.56 | Reduction |
| PPT_R | 49 | 28.41 ± 6.95 | -0.44 | Reduction |
| SLF_L | 9 | 23.11 ± 2.13 | -0.90 | Reduction |
| SLF_R | 2 | 20.00 ± 0 | -0.28 | Reduction |
| TPT_R | 1 | 20.00 ± 0 | -0.40 | Reduction |
| UF_L | 26 | 29.00 ± 5.30 | -2.82 | Reduction |
| all bundles | 2077 | 32.54 ± 10.61 | -2.30 | Reduction |
| CC | 37 | 23.95 ± 3.90 | 0.33 | Increase |
| CS_L | 77 | 23.69 ± 3.58 | 1.12 | Increase |
| CS_R | 1 | 20.00 ± 0 | 0.15 | Increase |
| CST_L | 76 | 24.29 ± 4.54 | 0.94 | Increase |
| CST_R | 2 | 20.00 ± 0 | 0.14 | Increase |
| CT_L | 24 | 23.5 ± 4.21 | 0.39 | Increase |
| EMC_L | 14 | 23.29 ± 1.62 | 0.84 | Increase |
| FAT_L | 1 | 20.00 ± 0 | 0.19 | Increase |
| FPT_L | 52 | 23.85 ± 4.29 | 1.12 | Increase |
| FPT_R | 2 | 20.00 ± 0 | 0.14 | Increase |
| PPT_L | 81 | 25.01 ± 4.79 | 0.92 | Increase |
| PPT_R | 2 | 20.00 ± 0 | 0.14 | Increase |
| all bundles | 369 | 24.04 ± 4.24 | 0.87 | Increase |

**Abbreviations**: *AC*: Anterior Commissure; *AF*: Arcuate Fasciculus ; *AR*: Acoustic Radiation; *C*: Cingulum; *CC*: Corpus Callosum; *CS*: Corticostriatal Pathway; *CST*: Corticospinal Tract; *CT*: Corticothalamic Pathway; *EMC*: Extreme Capsule; *F*: Fornix; *FPT*: Frontopontine Tract; *IFOF*: Inferior Fronto Occipital Fasciculus; *ILF*: Inferior Longitudinal Fasciculus; *MdLF*: Middle Longitudinal Fasciculus; *OPT*: Occipitopontine Tract; *OR*: Optic Radiation; *PPT*: Parietopontine Tract; *SLF*: Superior Longitudinal Fasciculus; *TPT*: Temporopontine Tract; *UF*: Uncinate Fasciculus; *FAT*: Frontal Aslant Tract. L – Left; R – Right.

**Table S5.** **White matter changes significantly different  between right TLE patients and controls 3 months after surgery.** The subsections were grouped in the set of bundles of the connectome atlas. Cohen’s d between groups, number of altered streamlines (No streamlines) and mean length for each bundle is presented below. The Cohen’s d between groups taking into account all the subsections found by connectometry without any grouping is also presented with the label “all bundles”.

| Bundles | No streamlines | Mean streamline length (± standard deviation) | cohen-D | QA Finding |
| --- | --- | --- | --- | --- |
| AC | 255 | 35.21 ± 10.53 | -3.63 | Reduction |
| AF_L | 2 | 21.00 ± 1.00 | -0.58 | Reduction |
| AF_R | 37 | 25.51 ± 8.78 | -1.30 | Reduction |
| C_L | 5 | 23.60 ± 2.94 | -0.86 | Reduction |
| C_R | 119 | 33.98 ± 11.49 | -0.81 | Reduction |
| CC | 650 | 27.77 ± 6.36 | -1.19 | Reduction |
| CS_L | 14 | 22.00 ± 2.39 | -0.64 | Reduction |
| CS_R | 61 | 22.56 ± 2.41 | -1.95 | Reduction |
| CST_L | 4 | 26.50 ± 2.18 | -0.73 | Reduction |
| CST_R | 136 | 24.93 ± 3.61 | -1.76 | Reduction |
| CT_L | 12 | 24.83 ± 7.09 | -0.78 | Reduction |
| CT_R | 198 | 26.55 ± 6.35 | -3.12 | Reduction |
| EMC_R | 6 | 22.00 ± 1.15 | -2.32 | Reduction |
| F_L | 3 | 23.33 ± 3.40 | -1.04 | Reduction |
| F_R | 76 | 27.84 ± 5.91 | -2.34 | Reduction |
| FAT_L | 5 | 22.40 ± 1.50 | -0.96 | Reduction |
| FAT_R | 33 | 21.58 ± 1.37 | -1.71 | Reduction |
| FPT_L | 17 | 23.53 ± 2.52 | -0.99 | Reduction |
| FPT_R | 85 | 23.34 ± 2.78 | -2.38 | Reduction |
| IFOF_R | 513 | 34.41 ± 10.59 | -3.22 | Reduction |
| ILF_R | 8 | 36.25 ± 10.51 | -1.88 | Reduction |
| MdLF_R | 50 | 24.64 ± 3.16 | -1.55 | Reduction |
| OPT_R | 31 | 24.13 ± 2.87 | -1.60 | Reduction |
| OR_R | 2 | 23.00 ± 1.00 | -0.72 | Reduction |
| PPT_L | 6 | 26.00 ± 4.76 | -0.57 | Reduction |
| PPT_R | 89 | 25.44 ± 3.60 | -1.50 | Reduction |
| SLF_R | 70 | 23.31 ± 3.45 | -1.80 | Reduction |
| U_R | 6 | 22.33 ± 2.13 | -1.54 | Reduction |
| UF_R | 100 | 35.78 ± 10.38 | -4.97 | Reduction |
| all bundles | 2593 | 29.37 ± 9.04 | -2.76 | Reduction |

**Abbreviations**: *AC*: Anterior Commissure; *AF*: Arcuate Fasciculus ; *AR*: Acoustic Radiation; *C*: Cingulum; *CC*: Corpus Callosum; *CS*: Corticostriatal Pathway; *CST*: Corticospinal Tract; *CT*: Corticothalamic Pathway; *EMC*: Extreme Capsule; *F*: Fornix; *FAT*: Frontal Aslant Tract; *FPT*: Frontopontine Tract; *IFOF*: Inferior Fronto Occipital Fasciculus; *ILF*: Inferior Longitudinal Fasciculus; *MdLF*: Middle Longitudinal Fasciculus; *OPT*: Occipitopontine Tract; *OR*: Optic Radiation; *PPT*: Parietopontine Tract; *SLF*: Superior Longitudinal Fasciculus; *U*: U-fiber; *UF*: Uncinate Fasciculus. L – Left; R – Right.

**Table S6.** **White matter changes significantly different between left TLE patients with different surgical outcomes over 5 years.** The subsections were grouped in the set of bundles of the connectome atlas. Cohen’s d between groups, number of altered streamlines (No streamlines) and mean length for each bundle is presented below. The Cohen’s d between groups taking into account all the subsections found by connectometry without any grouping is also presented with the label “all bundles”.

| Bundles | No streamlines | Mean streamline length (± standard deviation) | Cohen’s d | QA Finding |
| --- | --- | --- | --- | --- |
| AC | 17 | 24.82 ± 2.99 | -1.14 | Reduction |
| AF_R | 3 | 27.33 ± 5.25 | -0.18 | Reduction |
| C_L | 6 | 23.67 ± 3.35 | -0.66 | Reduction |
| CC | 263 | 25.29 ± 6.08 | -0.29 | Reduction |
| CS_L | 13 | 25.09 ± 5.22 | -0.86 | Reduction |
| CST_R | 26 | 22.77 ± 2.89 | -0.03 | Reduction |
| CT_R | 25 | 22.63 ± 2.34 | -0.08 | Reduction |
| EMC_L | 1 | 21.71 ± 0.70 | -1.92 | Reduction |
| IFOF_L | 137 | 24.00 ± 4.08 | -2.93 | Reduction |
| IFOF_R | 12 | 22.75 ± 3.53 | -0.20 | Reduction |
| ILF_L | 1 | 22.88 ± 2.27 | -0.37 | Reduction |
| ILF_R | 1 | 26.00 ± 0 | -0.17 | Reduction |
| OPT_R | 4 | 23.00 ± 1.73 | -0.08 | Reduction |
| OR_R | 3 | 22.38 ± 2.37 | -0.01 | Reduction |
| PPT_R | 31 | 30.60 ± 7.57 | -0.06 | Reduction |
| SLF_L | 5 | 24.00 ± 2.83 | -0.23 | Reduction |
| SLF_R | 3 | 20.00 ± 0 | -0.10 | Reduction |
| U_R | 1 | 20.00 ± 0 | -0.17 | Reduction |
| UF_L | 1 | 22.00 ± 0 | -1.96 | Reduction |
| UF_R | 1 | 21.50 ± 1.66 | -0.10 | Reduction |
| all bundles | 554 | 23.33 ± 1.89 | -1.24 | Reduction |

**Abbreviations**: *AC*: Anterior Commissure; *AF*: Arcuate Fasciculus; *C*: Cingulum; *CC*: Corpus Callosum; *CS*: Corticostriatal Pathway; *CST*: Corticospinal Tract; *CT*: Corticothalamic Pathway; *EMC*: Extreme Capsule; *F*: Fornix; *FPT*: Frontopontine Tract; *IFOF*: Inferior Fronto Occipital Fasciculus; *ILF*: Inferior Longitudinal Fasciculus; *OPT*: Occipitopontine Tract; *OR*: Optic Radiation; *PPT*: Parietopontine Tract; *SLF*: Superior Longitudinal Fasciculus; *U*: U-fiber; *UF*: Uncinate Fasciculus. L – Left; R – Right.

**Table S7.** AAL regions and their abbreviation included in the study.

| Regions | Abbr. |
| --- | --- |
| Precental gyrus | PreC |
| Superior frontal gyrus, dorsolateral | SF |
| Superior frontal gyrus, orbital part | ORBsup |
| Middle frontal gyrus | MF |
| Middle frontal gyrus, orbital part | ORBmid |
| Inferior frontal gyrus, opercular part | IFOper |
| Inferior frontal gyrus, triangular part | IFTrian |
| Inferior frontal gyrus, orbital part | ORBinf |
| Rolandic operculum | ROL |
| Supplementary motor area | SMA |
| Olfactory cortex | OLF |
| Superior frontal gyrus, medial | SFMed |
| Superior frontal gyrus, medial orbital | ORBsupmed |
| Gyrus rectus | REC |
| Insula | INS |
| Anterior cingulate and paracingulate gyri | AC |
| Median cingulate and paracingulate gyri | MC |
| Posterior cingulate gyrus | PC |
| Hippocampus | HIP |
| Parahippocampal gyrus | PHIP |
| Amygdala | AMY |
| Calcarine fissure and surrounding cortex | CAL |
| Cuneus | CUN |
| Lingual gyrus | LIN |
| Superior occipital gyrus | SO |
| Middle occipital gyrus | MO |
| Inferior occipital gyrus | IO |
| Fusiform gyrus | FFG |
| Postcentral gyrus | PoC |
| Superior parietal gyrus | SP |
| Inferior parietal, but supramarginal and angular gyri | IP |
| Supramarginal gyrus | SMG |
| Angular gyrus | ANG |
| Precuneus | PCUN |
| Paracentral lobule | PCL |
| Caudate nucleus | CAU |
| Lenticular nucleus, putamen | PUT |
| Lenticular nucleus, pallidun | PAL |
| Thalamus | THA |
| Heschl gyrus | HES |
| Superior temporal gyrus | ST |
| Temporal pole: superior temporal gyrus | TPOsup |
| Middle temporal gyrus | MT |
| Temporal pole: middle temporal gyrus | TPOmid |
| Inferior temporal gyrus | IT |

*Note:* table adapted from Liu *et al.,* 2014.

**Table S8.** Tracts from the connectome atlas and their abbreviations.

| Classification | Bundles | Abbr. |
| --- | --- | --- |
| Association | Arcuate Fasciculus | AF |
|  | Cingulum | C |
|  | Extreme Capsule | EMC |
|  | Frontal Aslant Tract | FAT |
|  | Inferior Fronto Occipital Fasciculus | IFOF |
|  | Inferior Longitudinal Fasciculus | ILF |
|  | Middle Longitudinal Fasciculus | MdLF |
|  | Superior Longitudinal Fasciculus | SLF |
|  | U-fiber | U |
|  | Uncinate Fasciculus | UF |
|  | Vertical Occipital Fasciculus | VOF |
| Commissural | Anterior Commissure | AC |
|  | Corpus Callosum | CC |
|  | Posterior Commissure | PC |
| Projection | Acoustic Radiation | AR |
|  | Corticostriatal Pathway | CS |
|  | Corticospinal Tract | CST |
|  | Corticothalamic Pathway | CT |
|  | Fornix | F |
|  | Frontopontine Tract | FPT |
|  | Occipitopontine Tract | OPT |
|  | Optic Radiation | OR |
|  | Parietopontine Tract | PPT |
|  | Temporopontine Tract | TPT |

## **Supplementary Figures**

**Figure S1. QA Changes over 3 months between controls and left TLE patients.** We ran connectometry analysis with different t-score thresholds to select local connectomes (T-score: 1, 2 and 3) at different significance levels (FDR: 0.05, 0.075 and 0.1). Higher values of t-score give more specific and confirmatory results, whereas lower values give more sensitive results, which are useful for exploratory studies. The square with black borders indicates the grid search point selected for further analysis in the study.

**Figure S2. QA Changes over 3 months between controls and right TLE patients.** We ran connectometry analysis with different t-score thresholds to select local connectomes (T-score: 1, 2 and 3) at different significance levels (FDR: 0.05, 0.075 and 0.1). Higher values of t-score give more specific and confirmatory results, whereas lower values give more sensitive results, which are useful for exploratory studies. The square with black borders indicates the grid search point selected for further analysis in the study.


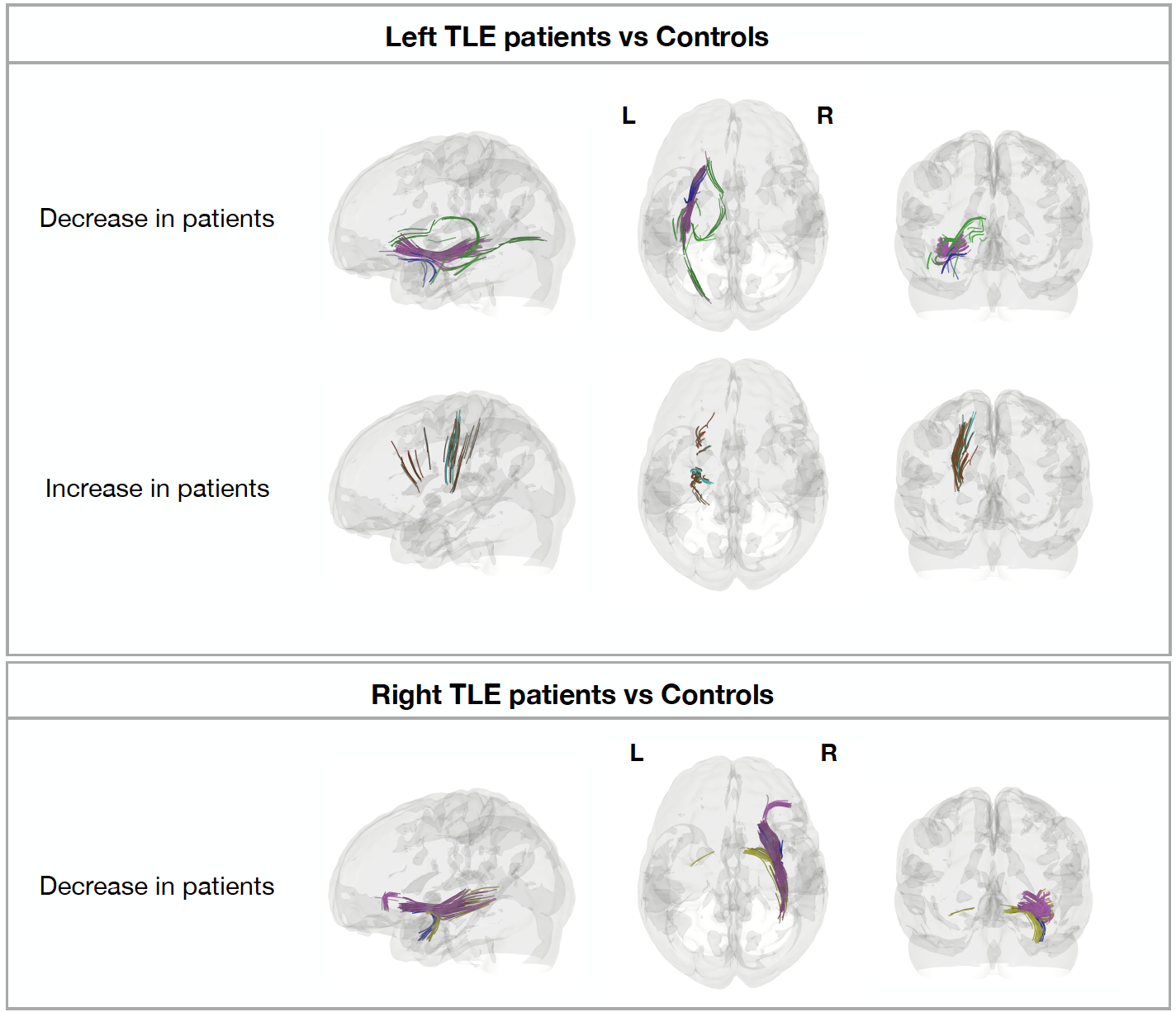


**Figure S3. Subsections of the bundles with greater changes over 3 months in TLE patients.** For left TLE patients, greater reductions were seen in ipsilateral inferior fronto-occipital fasciculus (IFOF), uncinate fasciculus (UF) and corticothalamic pathway (CT). Left TLE patients also showed greater increase in QA over 3-4 months in ipsilateral corticostriatal pathway (CS), frontopontine tract (FPT) and corticospinal tract (CST). Right TLE patients showed greater reduction in IFOF, UF and anterior commissure (AC). In both left and right patients, the affected subsections in IFOF extend to the frontal cortex superiorly to UF. Purple – IFOF; Blue – UF; Green – CT, Cyan - CST; Brown - CS ; Red – FPT; Yellow – AC. L - Left; R - Right.


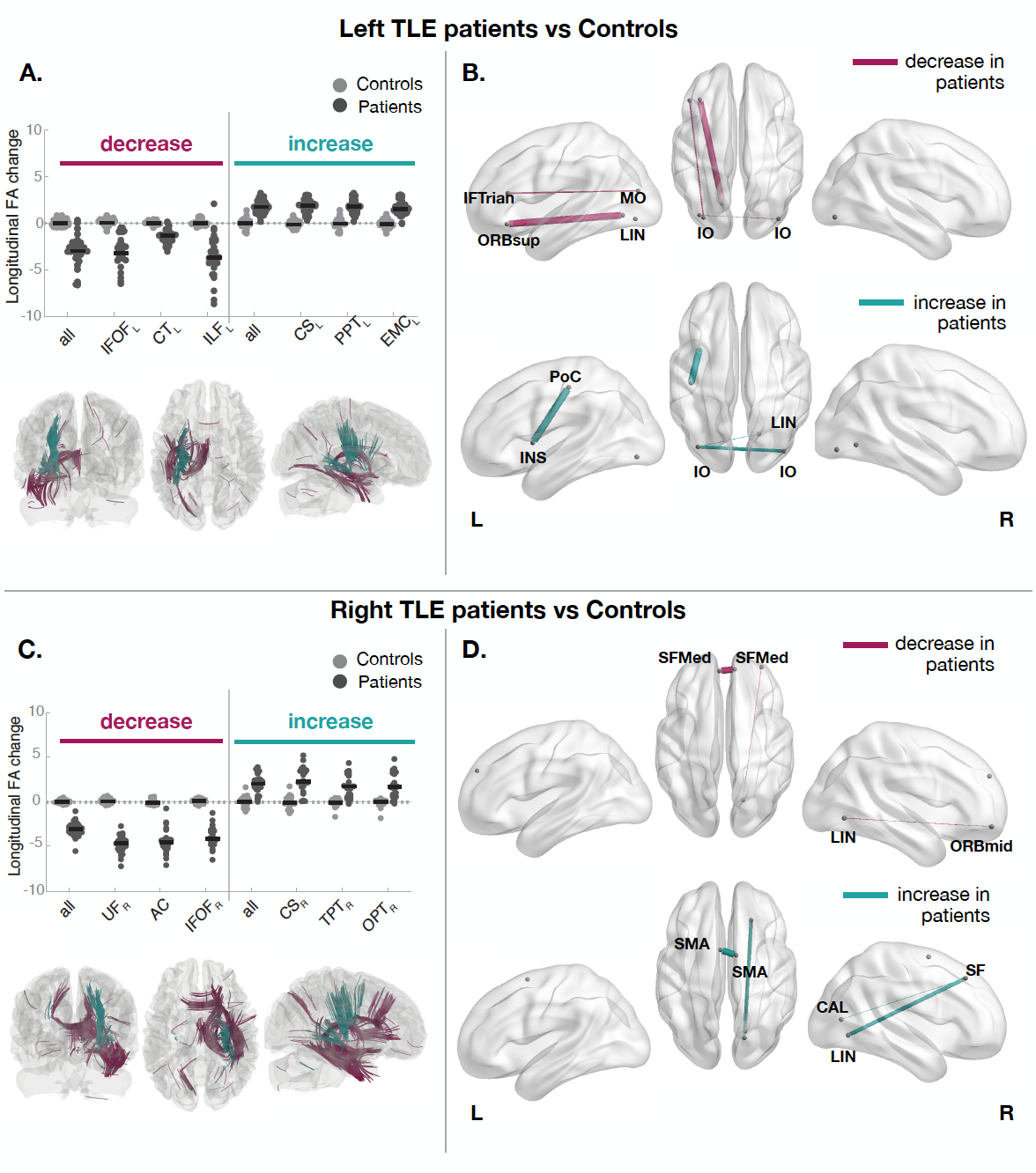


**Figure S4. Significant FA changes between controls and TLE patients, before and after surgery, in parts of tracts revealed by connectometry.** Significant FA changes in subsections of bundles were found between groups (FDR<0.05; T-score=3). Subsections of tracts in which patients had a significant FA reduction or increase relative to controls are coloured in purple and green, respectively **(A,C)**. Left and right TLE patients had a greater decrease of FA in ipsilateral inferior fronto-occipital fasciculus (IFOF). Greater reductions in FA were also observed in ipsilateral corticothalamic pathway (CT) and inferior longitudinal fasciculus (ILF) in left TLE patients. Right TLE patients also showed greater reduction in anterior commissure (AC) and uncinate fasciculus (UF). Left TLE showed a greater increase of FA in ipsilateral corticostriatal pathway (CS), parietopontine tract (PPT) and extreme capsule (EMC). Right TLE showed a greater increase in CS, temporopontine (TPT) and occipitopontine (OPT). The beeswarm plots show the longitudinal changes in FA between groups for all subsections found by connectometry (label *all* in the plot) and the bundles with greater difference between groups. Each datapoint indicates a single subject. For visualisation purposes, only the three bundles with highest values of cohen-D are presented in the beeswarm plot. **(B,D)** The tract bundles where subsections were found to be significantly different by connectometry were determined to estimate the AAL regions those subsections are connecting to. The weight of the connections/edges is proportional to the amount of tracts with altered subsections. For visualisation, only the top 70% of strongest connections are shown. A table with the names and abbreviations of all regions and bundles can be found in Tables S7 and S8.

Cal - *Calcarine*; ORBsup - *Superior frontal, orbital;*IFTrian - *Inferior frontal, triangular;* IO - *Inferior occipital;* INS - *Insula;* LIN - *Lingual*; ORBmid - *Middle frontal, orbital;* MO - *Middle occipital;* SF - *Superior frontal, dorsolateral;* SFMed - *Superior frontal, medial;* SMA - *Supplementary Motor Area.* PoC – *Postcentral.*

**
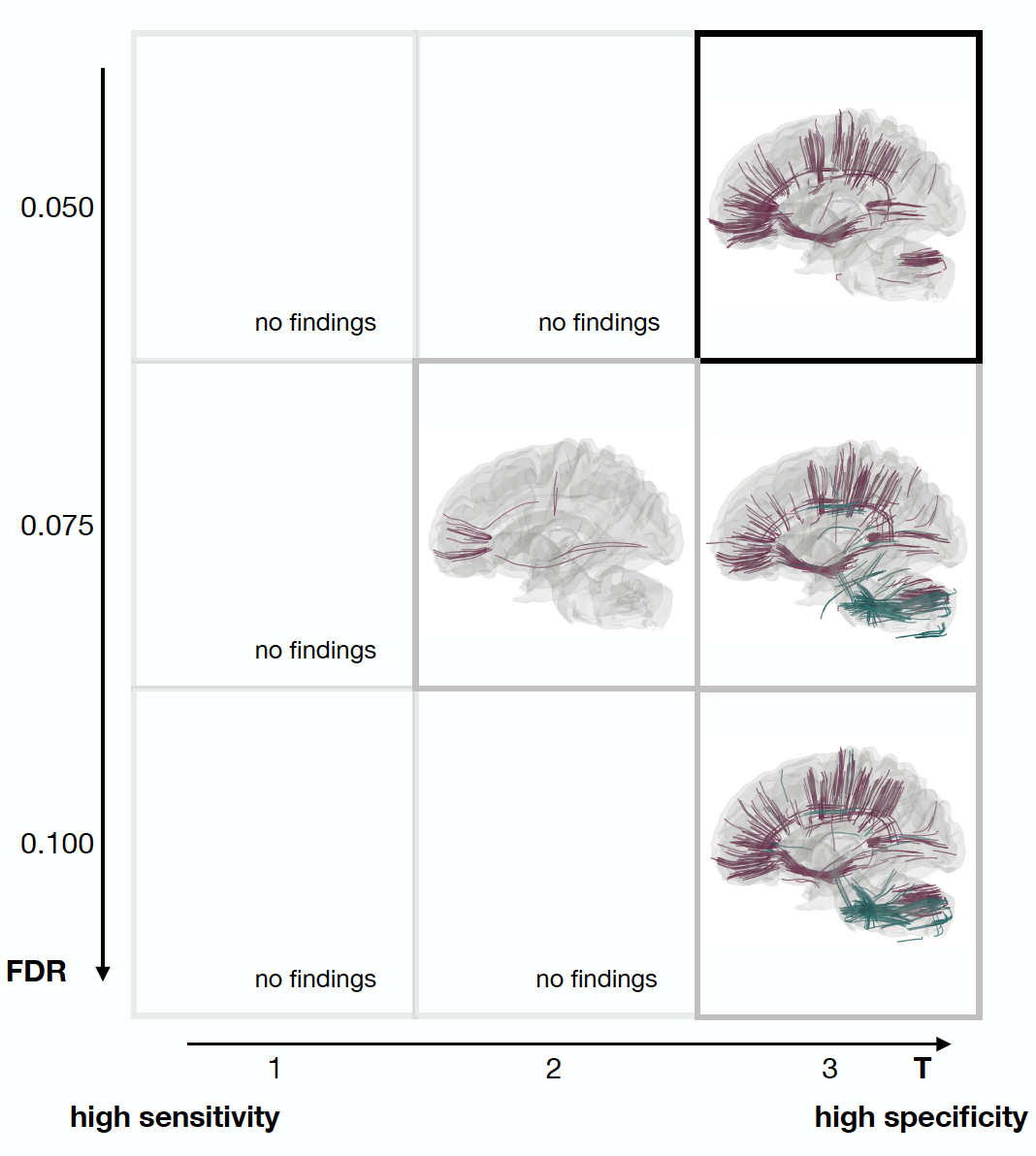
**

**Figure S5 – QA Changes over 3 months between left TLE patients seizure-free over 5 years and left TLE patients with relapse.** We ran connectometry analysis with different t-score thresholds to select local connectomes (T-score: 1, 2 and 3) at different significance levels (FDR: 0.05, 0.075 and 0.1). Higher values of t-score give more specific and confirmatory results, whereas lower values give more sensitive results, which are useful for exploratory studies. The square with black borders indicates the grid search point selected for further analysis in the study.


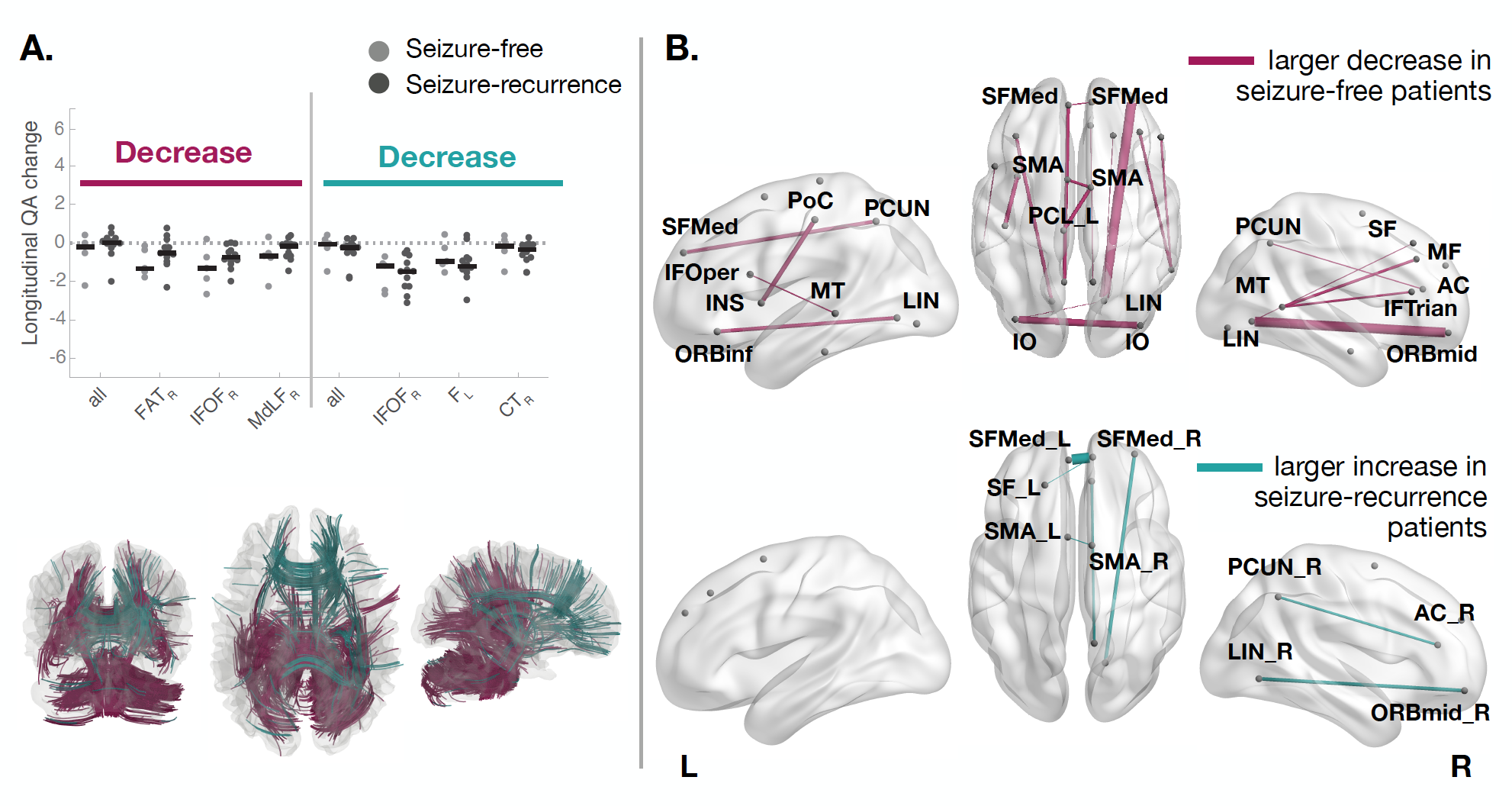


**Figure S6. Tract sections with QA changes over 3 months that are associated with postoperative seizure freedom in patients with right TLE.** Significant QA changes in subsections of bundles were found between outcome groups (FDR < 0.05; T-score = 3). **(A)** Subsections of tracts where seizure-free patients had a significantly larger QA reduction over 3 months in comparison to patients with seizure-recurrence are coloured in purple. Subsections of tracts where seizure-recurrence patients had a significantly larger QA reduction over 3 months in comparison to seizure-free patients are coloured in green. The beesswarm plot shows the longitudinal changes in QA between groups for all subsections found by connectometry (label *all* in the plot) and the bundles with greater reductions. For visualisation purposes, only the three bundles with the highest Cohen’s d are displayed (Table S7 contains complete list). **(B)** The tract bundles where subsection were found to be significant different by connectometry were determined to estimate the AAL regions those subsections are connecting to. The weight of the connections/edges is proportional to the amount of tracts with altered subsections. For visualisation purposes, a threshold was applied to hide the edges with a weight less than 30% of the strongest connection. A table with the names and abbreviations of all regions and bundles can be found in Tables S7 and S8.


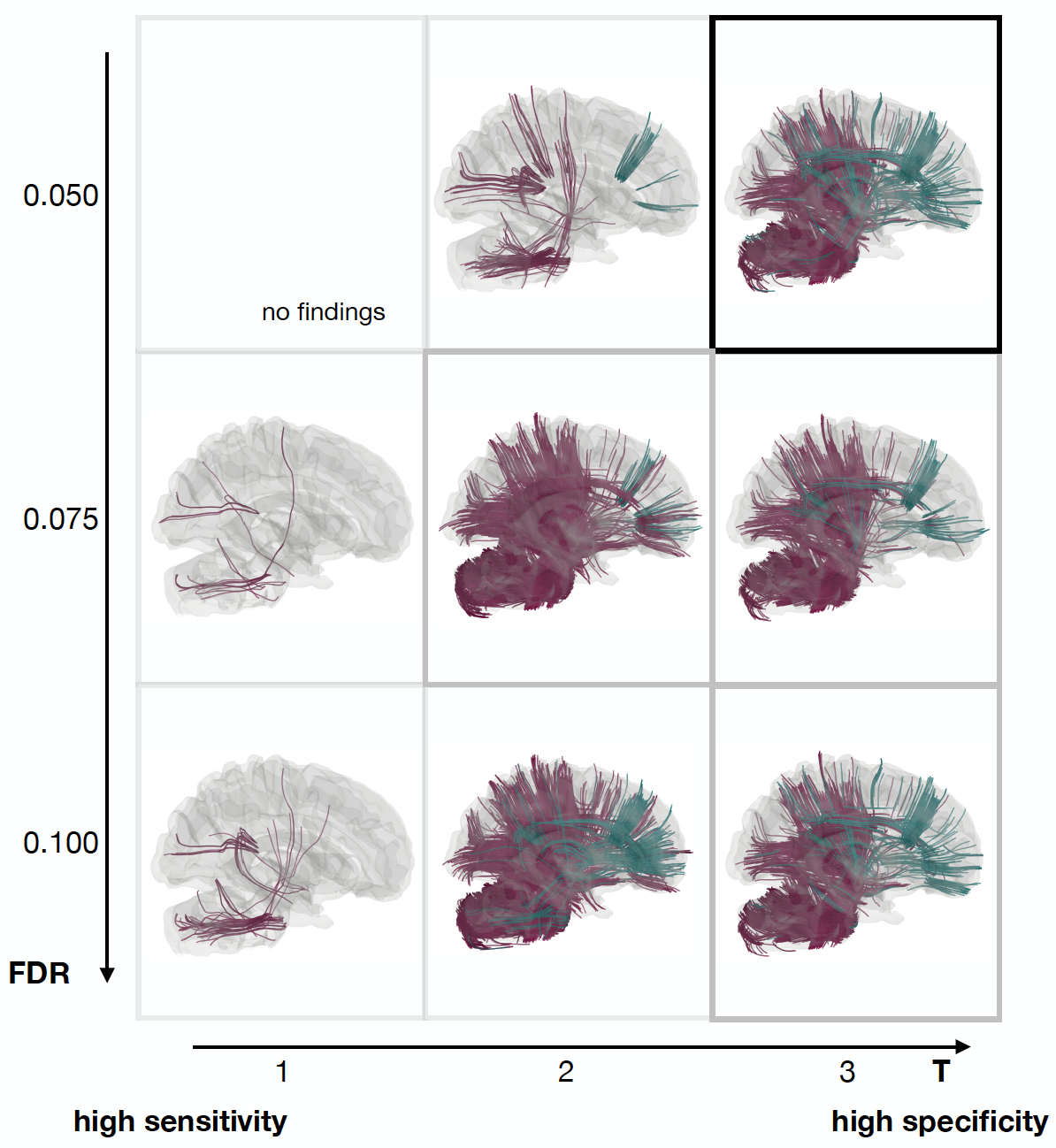


**Figure S7 –QA Changes over 3 months between right TLE patients seizure-free over 5 years and right TLE patients with relapse.** We ran connectometry analysis with different t-score thresholds to select local connectomes (T-score: 1, 2 and 3) at different significance levels (FDR: 0.05, 0.075 and 0.1). Higher values of t-score give more specific and confirmatory results, whereas lower values give more sensitive results, which are useful for exploratory studies. The square with black borders indicates the grid search point selected for further analysis in the study.


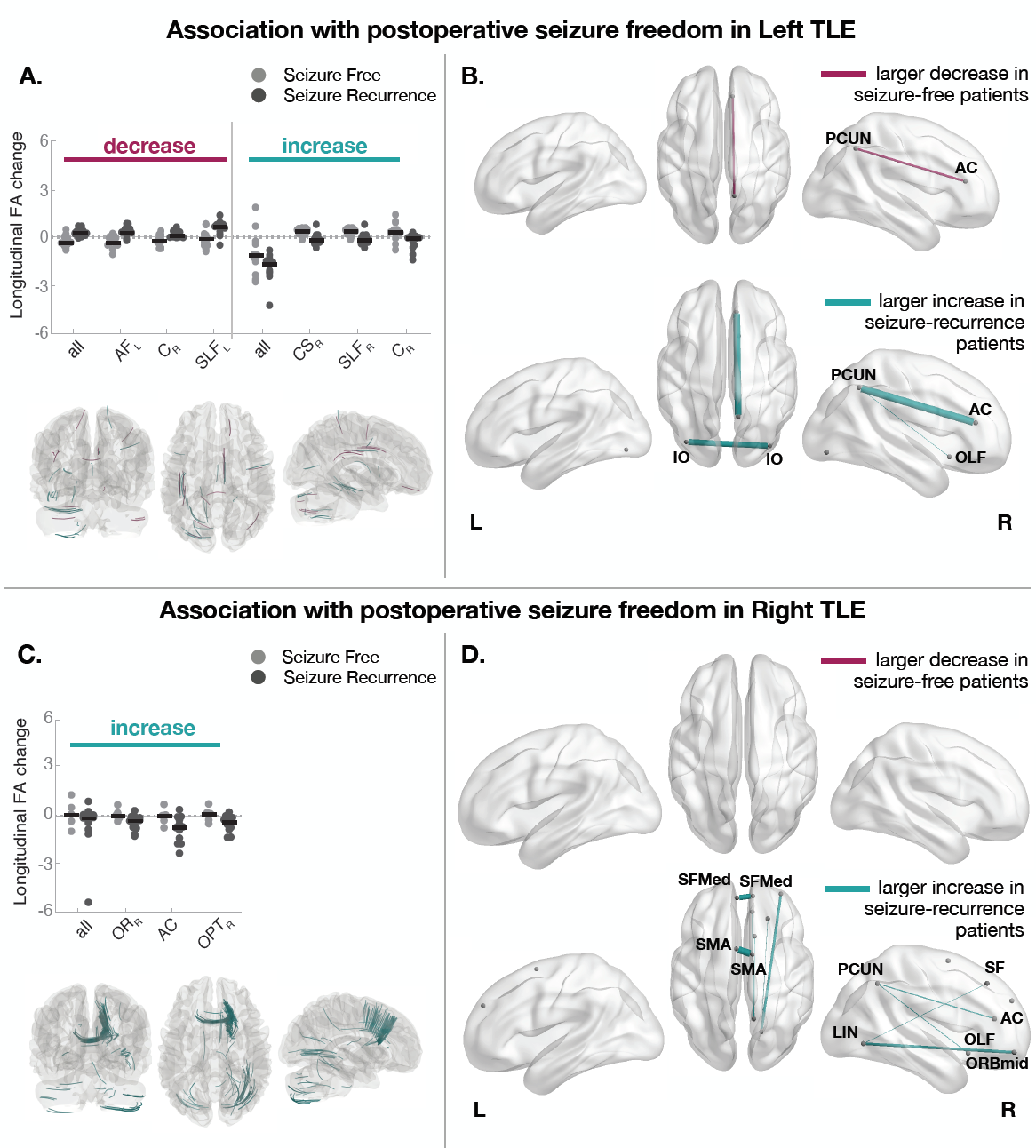


**Figure S8. Tract sections with FA changes over 3 months that are associated with postoperative seizure freedom in patients with left and right TLE.** Significant FA changes in subsections of bundles were found between outcome groups (FDR < 0.05; T-score = 3). **(A)** Subsections of tracts where seizure-free patients had a significantly larger FA reduction over 3 months in comparison to patients with seizure-recurrence are coloured in purple. Subsections of tracts where seizure-recurrence patients had a significantly larger FA reduction over 3 months in comparison to seizure-free patients are coloured in green. The beesswarm plot shows the longitudinal changes in QA between groups for all subsections found by connectometry (label *all* in the plot) and the bundles with greater reductions. For visualisation purposes, only the three bundles with the highest Cohen’s d are displayed. **(B)** The tract bundles where subsection were found to be significant different by connectometry were determined to estimate the AAL regions those subsections are connecting to. The weight of the connections/edges is proportional to the amount of tracts with altered subsections. For visualisation purposes, a threshold was applied to hide the edges with a weight less than 30% of the strongest connection. A table with the names and abbreviations of all regions and bundles can be found in Tables S7 and S8.

**
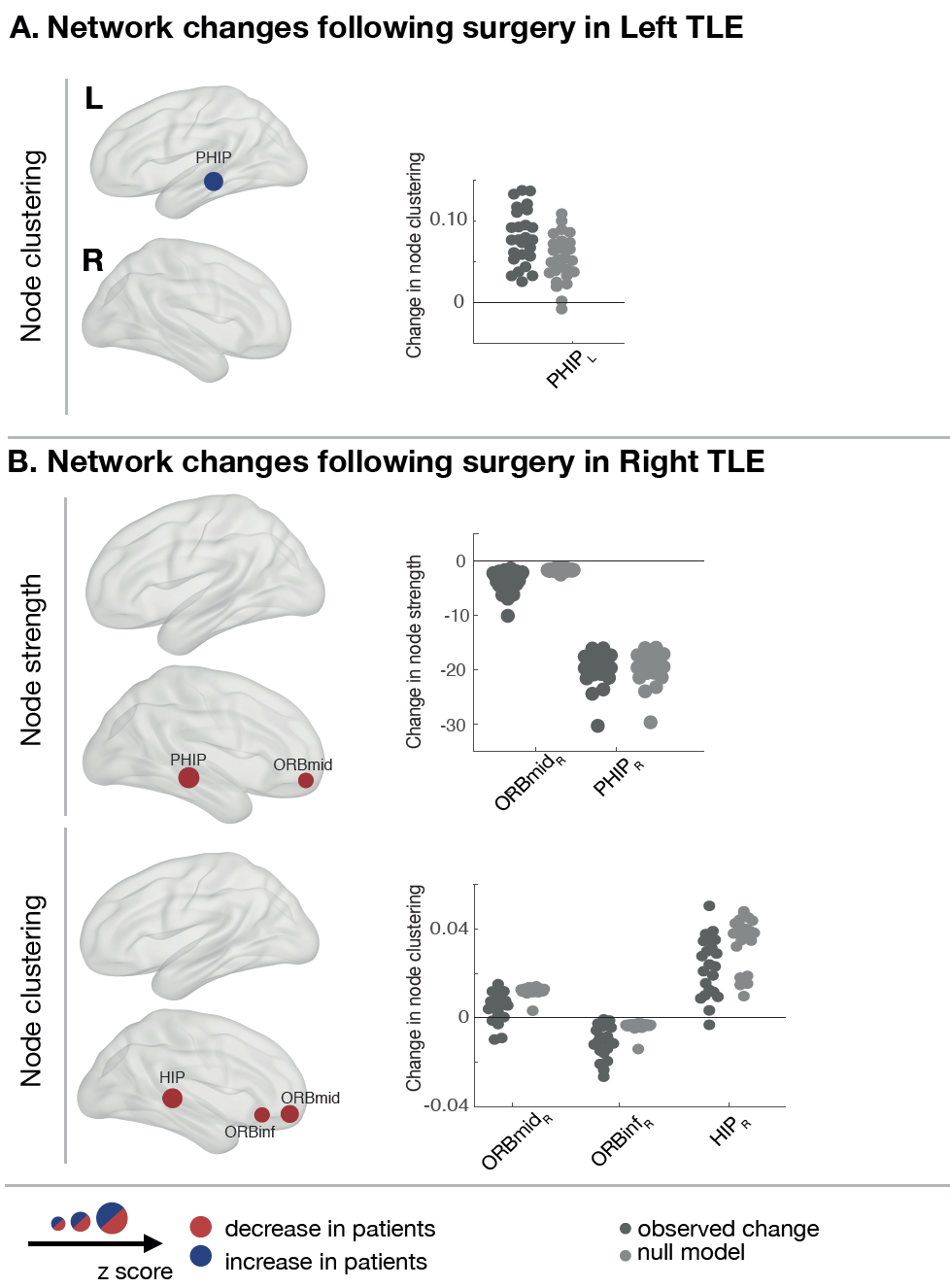
**

**Figure S9. Changes over 3 months in node clustering and node strength patients and controls.** The AAL regions that showed significant differences in node clustering and node strength between patients and controls over 3 months are represented by a node/circle and overlaid within a brain volume. Nodes coloured in blue show the AAL regions where patients had an increase in node clustering in comparison to controls over 3 months. Nodes coloured in red show the AAL regions in which patients had a significant reduction in strength or clustering in comparison to controls. Scatter plots show the observed and predicted (null model) change in patients after surgery for those AAL regions. Positive values suggest an increase of node strength or clustering following surgery. The scatter plots therefore compare the impact on network measures of the surgical resection alone with the overall changes seen post-operatively. No significant differences in node efficiency were observed between patients and controls A table with the names and abbreviations of all regions can be found in Table S8.

HIP – *Hippocampus;* ORBinf - *Inferior frontal, orbital*; ORBmid - *Middle frontal, orbital*; PHIP – *Parahippocampal*.


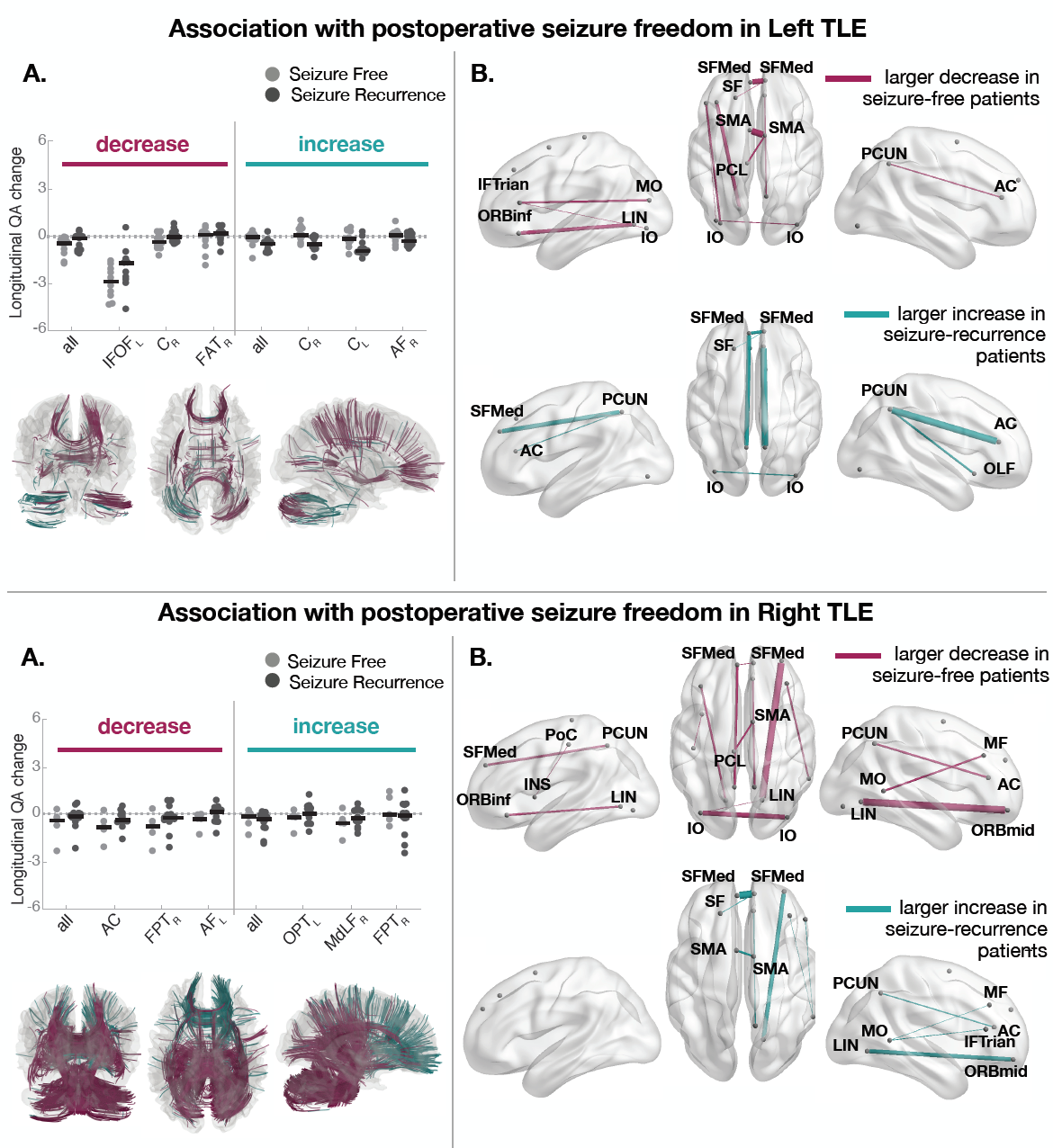


**Figure S10. Tract sections with QA changes over 3 months that are associated with postoperative seizure freedom in patients with left and right TLE after regressing out the resection volume.** Significant QA changes in subsections of bundles were found between outcome groups (FDR < 0.05; T-score = 3). **(A)** Subsections of tracts where seizure-free patients had a significantly larger QA reduction over 3 months in comparison to patients with seizure-recurrence are coloured in purple. Subsections of tracts where seizure-recurrence patients had a significantly larger QA reduction over 3 months in comparison to seizure-free patients are coloured in green. The beesswarm plot shows the longitudinal changes in QA between groups for all subsections found by connectometry (label *all* in the plot) and the bundles with greater reductions. For visualisation purposes, only the three bundles with the highest Cohen’s d are displayed. **(B)** The tract bundles where subsection were found to be significant different by connectometry were determined to estimate the AAL regions those subsections are connecting to. The weight of the connections/edges is proportional to the amount of tracts with altered subsections. For visualisation purposes, a threshold was applied to hide the edges with a weight less than 30% of the strongest connection. A table with the names and abbreviations of all regions and bundles can be found in Tables S7 and S8.

**Figure S10. Reduced QA after surgery over 3 months.** Bundles from the connectome atlas which showed statistical significance after FDR correction between groups over 3 months **(A,C)** and over 12 months **(B,D)**. Longitudinal QA change is the difference between post-operative and pre-operative mean QA after regressing out age and sex for left TLE **(A,B)** and right TLE **(C,D)**. Each data point represents a subject and the black line the median across subjects. **(E)** Location of the tracts. F – Fornix (green); IFOF – Inferior fronto occipital fasciculus (lilac); UF – Uncinate fasciculus (blue); L- Left; R – Right.

# **Supplementary Methods:**

## Imaging protocols:

All participants were presented for MRI scanning on a 3T GE Signa HDx scanner (General Electric, Waukesha, Milwaukee, WI) using a body coil for transmission and 8-channel phased array coil for reception. Standard imaging gradients with a maximum strength of 40 mTm-1 and slew rate 150 Tm-1s-1 were used. The study protocol consisted of a coronal 3D T1-weighted volumetric acquisition (matrix, 256×256×170; in-plane resolution, 0.9375×0.9375×1.1 mm) and diffusion weighted images (b-value =1200 s mm^-2^ [δ=21ms, ∆ =29 ms using full gradient strength of  40 mTm-1], 60 axial slices with 2.4mm thickness, 52 noncollinear directions, 6 non-diffusion weighted scans). Diffusion MRI data were acquired using a cardiac-triggered single-shot spin-echo planar imaging sequence (Wheeler-Kingshott et al., 2002) with echo time = 73 ms. The gradient directions were estimated and ordered as described in Cook et al., 2007. The field of view was 24 x 24 cm and the acquisition matrix size was 96×96, zero filled to 128×128 during reconstruction, resulting in a voxel size of 1.875 × 1.875 × 2.4 mm. These imaging protocols are identical to our previous study (Taylor et al. 2018).

## Atlas-based approach: Diffusion changes in whole white matter bundles

To investigate white matter alterations after resective surgery and their association with postoperative seizure-freedom, we employed an atlas-based/whole-tract approach to investigate changes in entire fasciculi. In comparison to connectometry, this approach is less sensible to focal changes within bundles. Nevertheless it is commonly employed by its simplicity and low computational cost.

White matter degeneration in the vicinity of the resected tissue may adversely affect fiber tractography-based approaches, leading to false negative connections. Therefore, the population-average atlas of the connectome (Yeh *et al.*, 2018) was preferred. This atlas identifies 550,000 white matter tracts verified manually by experienced neuroanatomists (Yeh *et al.*, 2018), which are grouped in 80 bundles. Cranial nerves, cerebellum and brainstem bundles were not considered in this study.

For each subject and timepoint, we estimated the mean QA for each bundle. We constrained the analysis to the non-resected area using the resection masks. After obtaining the mean QA for each participant and timepoint, we computed the longitudinal change over 3-4 months and over the following 8-9 months.  We compared the longitudinal changes between patient and control groups using inference statistical tests.

To study the association of longitudinal changes with postoperative seizure-freedom, we grouped patients in 2 classes as follows: Class 1:Seizure-free, if the patient remained ILAE1 throughout the 5 years of follow up, and class 2:Seizure-recurrence, if the patient had ILAE>1 at any time during the five-years follow up. Longitudinal changes between both groups or classes were compared using inference statistical tests.

## Group subsections from connectometry

The subsections of tracts found to be significantly different between patients and controls were grouped as in the population-average atlas of the connectome (Yeh *et al.*, 2018, which comprise 550,000 white matter tracts grouped in 80 bundles. Cranial nerves, cerebellum and brainstem bundles were not considered in this study. We imported the coordinates of the subsections of tracts found by connectometry and the tracts of each bundle of the connectome atlas into MATLAB. Only subsections at 1mm or less from a tract in the connectome atlas were considered. Any other subsections were considered as false positives.

As in the connectome atlas, the subsections were grouped and labelled by the bundle name of the closest tract of the connectome atlas.

To study what grey matter regions these subsections of tracts were connecting, we estimate their entire length. To this purpose, we saved the closest tract of the connectome atlas to each subsection. After estimating the entire length of the subsections, we computed a connectivity matrix using AAL parcellation scheme. The area of the AAL regions overlapping with the resection tissue was labelled as a new ROI termed RT.

# **Supplementary Results:**

## Atlas-based approach: Diffusion changes in white matter bundles

A white matter atlas-based approach was performed to identify alterations in connectome atlas’ bundles. Surgery-induced changes in white matter were characterised by comparing longitudinal QA changes between TLE patients and controls over 3-4 months after surgery and over the following 8-9 months. After temporal lobe resection, there were significant reductions in QA located in the ipsilateral fornix (F) (Left TLE: FDR p-value < 0.001; Right TLE: FDR p-value = 0.005) and uncinate fasciculus (UF) (Left TLE: FDR p-value = 0.047; Right TLE: FDR p-value = 0.001) (Figure S10). There was also a significant reduction of QA in the ipsilateral inferior fronto-occipital fasciculus (IFOF) for right TLE patients (FDR p-value =  0.035). As expected, controls showed longitudinal QA changes around zero over 3-4 months after surgery. No significant changes were found between 3 and 12 months after surgery between groups, including the bundles with significant reductions in QA over 3-4 months after surgery.

To investigate the association of surgery-induced changes in QA with postoperative seizure-freedom for each bundle, TLE patients were grouped based on whether they were seizure free or not. Longitudinal QA changes after the surgery were not significantly different between TLE patients with different outcomes (Ranksum test, p > 0.05 for all tract bundles).

## Association with postoperative outcome for right TLE patients

In right TLE, we found widespread differences between outcome groups in subsections of tracts (Figure S6). Similarly to left TLE patients, we found greater QA reductions relate to greater chances of postoperative seizure freedom in the vicinity of resection area, between lingual and orbitofrontal cortices and in interhemispheric connections in frontal and occipital lobes. Interhemispheric connections with reduction in QA associated with seizure-freedom were mostly seen in the occipital lobe and to a less extent in frontal lobe as opposed to left TLE. Nevertheless, we also observed greater reduction in some interhemispheric connections in frontal lobe associated to seizure-recurrence.

Although we observed significant changes over 3-4 months for right TLE patients, we caution against overinterpretation of this result given the small sample size, particularly for seizure-free group. Only 5 patients were seizure-free for the 5-year follow up period, which may not represent properly the variability within group.

## Consistency of resection masks

In order to verify if the resections were consistent across patients, the resective masks were inspected by visual inspection and Dice overlap was computed to quantity masks’ similarity. As described in Moreira da Silva *et al.* (2017) and Traynor *et al.* (2010), Dice overlap was computed for all possible pairs of masks across patients, as explained below:

$$overlap= \frac{\sum_{m} 2\alpha N(A\cap B)}{\sum_{m} \alpha(N\left( A \right)+N\left( B \right))}$$

where m represents every combination of pairs between patients, α the weight, N the number of voxels and A and B the resection masks of the pair m.

We used two different weights, 1, in which bigger masks contribute more to the similarity index and 1/V, in which masks contribute equally. V is the mean volume of number of voxels in A and B. As described in Table S2, the similarity of the resections masks across patients is high.

**Table S2.** **Similarity of the resection masks across patients.** The similarity was computed by Dice Overlap which ranges between 0 (no overlap) and 1 (full overlap). Both weighting approaches suggest a similar resection across patients with some variations, likely on the boundary of the resection.

| Weight | Left | Right |
| --- | --- | --- |
| 1 | 0.77 | 0.71 |
| 1/V | 0.74 | 0.72 |

We additionally investigated if resection volumes differed between groups. As expected, right temporal lobe epilepsy patients had larger resection volumes than left sided patients. These volumes were not significantly related to outcomes of seizure freedom however.

|  | Volume mean (standard deviation) | P value |
| --- | --- | --- |
| Left TLE volume (ILAE1/ILAE>1) | 50878 mm^3^ / 44294 mm^3^ (8769 mm^3^/ 14905 mm^3^) | 0.212 |
| Right TLE volume (ILAE1/ILAE>1) | 54630 mm^3^ / 47740 mm^3^ (6760 mm^3^/ 7370 mm^3^) | 0.104 |
